# MR-guided adaptive versus ITV-based stereotactic body radiotherapy for hepatic metastases (MAESTRO): a randomized controlled phase II trial

**DOI:** 10.1186/s13014-022-02033-2

**Published:** 2022-03-27

**Authors:** P. Hoegen, K. S. Zhang, E. Tonndorf-Martini, F. Weykamp, S. Regnery, P. Naumann, K. Lang, J. Ristau, S. A. Körber, C. Dreher, C. Buchele, C. Rippke, C. K. Renkamp, K. M. Paul, L. König, C. Büsch, J. Krisam, O. Sedlaczek, H.-P. Schlemmer, M. Niyazi, S. Corradini, J. Debus, S. Klüter, J. Hörner-Rieber

**Affiliations:** 1 Department of Radiation Oncology, Heidelberg University Hospital, Im Neuenheimer Feld 400, 69120 Heidelberg, Germany; 2 Heidelberg Institute of Radiation Oncology (HIRO), Heidelberg, Germany; 3 National Center for Tumor Diseases (NCT), Heidelberg, Germany; 4 Clinical Cooperation Unit Radiation Oncology, German Cancer Research Center (DKFZ), Heidelberg, Germany; 5 Division of Radiology, German Cancer Research Center (DKFZ), Heidelberg, Germany; 6 Department of Radiation Oncology, University Hospital Mannheim, Medical Faculty of Mannheim, University of Heidelberg, Mannheim, Germany; 7 Institute of Medical Biometry (IMBI), Heidelberg, Germany; 8 Department of Radiation Oncology, University Hospital, LMU Munich, Munich, Germany; 9 German Cancer Consortium (DKTK), Partner Site Munich, Munich, Germany; 10 Department of Radiation Oncology, Heidelberg Ion Beam Therapy Center (HIT), Heidelberg University Hospital, Heidelberg, Germany; 11 German Cancer Consortium (DKTK), Partner Site Heidelberg, Heidelberg, Germany

**Keywords:** Liver metastases, Stereotactic body radiotherapy (SBRT), MR-guided radiotherapy, MR-Linac, MRI, ITV, Adaptive radiotherapy (ART), Radiation-induced liver disease (RILD), Stereotactic ablative radiotherapy (SABR), Stereotactic Magnetic Resonance Guided Radiation Therapy (SMART)

## Abstract

**Background:**

Stereotactic body radiotherapy (SBRT) is an established local treatment method for patients with hepatic oligometastasis or oligoprogression. Liver metastases often occur in close proximity to radiosensitive organs at risk (OARs). This limits the possibility to apply sufficiently high doses needed for optimal local control. Online MR-guided radiotherapy (oMRgRT) is expected to hold potential to improve hepatic SBRT by offering superior soft-tissue contrast for enhanced target identification as well as the benefit of gating and daily real-time adaptive treatment. The MAESTRO trial therefore aims to assess the potential advantages of adaptive, gated MR-guided SBRT compared to conventional SBRT at a standard linac using an ITV (internal target volume) approach.

**Methods:**

This trial is conducted as a prospective, randomized, three-armed phase II study in 82 patients with hepatic metastases (solid malignant tumor, 1–3 hepatic metastases confirmed by magnetic resonance imaging (MRI), maximum diameter of each metastasis ≤ 5 cm (in case of 3 metastases: sum of diameters ≤ 12 cm), age ≥ 18 years, Karnofsky Performance Score ≥ 60%). If a biologically effective dose (BED) ≥ 100 Gy (α/β = 10 Gy) is feasible based on ITV-based planning, patients will be randomized to either MRgRT or ITV-based SBRT. If a lesion cannot be treated with a BED ≥ 100 Gy, the patient will be treated with MRgRT at the highest possible dose. Primary endpoint is the non-inferiority of MRgRT at the MRIdian Linac® system compared to ITV-based SBRT regarding hepatobiliary and gastrointestinal toxicity CTCAE III or higher. Secondary outcomes investigated are local, locoregional (intrahepatic) and distant tumor control, progression-free survival, overall survival, possible increase of BED using MRgRT if the BED is limited with ITV-based SBRT, treatment-related toxicity, quality of life, dosimetric parameters of radiotherapy plans as well as morphological and functional changes in MRI. Potential prognostic biomarkers will also be evaluated.

**Discussion:**

MRgRT is known to be both highly cost- and labor-intensive. The MAESTRO trial aims to provide randomized, higher-level evidence for the dosimetric and possible consecutive clinical benefit of MR-guided, on-table adaptive and gated SBRT for dose escalation in critically located hepatic metastases adjacent to radiosensitive OARs.

***Trial registration*:**

The study has been prospectively registered on August 30th, 2021: Clinicaltrials.gov, “Magnetic Resonance-guided Adaptive Stereotactic Body Radiotherapy for Hepatic Metastases (MAESTRO)”, NCT05027711.

**Supplementary Information:**

The online version contains supplementary material available at 10.1186/s13014-022-02033-2.

## Background

Standard therapy for patients with hepatic oligometastases is surgical resection. Hepatic metastasectomy has been performed for more than 3 decades with 5-year survival rates of 50–60% and up to 20% long-term survivors [1–4]. However, only 15–20% of patients with hepatic oligometastases are initially eligible for such a radical surgical approach due to an unresectable tumor location, inadequate hepatic reserve or other comorbidities [5, 6]. For the majority of patients who are not amenable for surgery, alternative liver-directed therapies are offered for providing local control and potentially superior overall survival, e.g. radiofrequency ablation, microwave ablation, transarterial chemoembolization, cryoablation, high-dose-rate brachytherapy or stereotactic body radiotherapy (SBRT) [7, 8].

Following hepatic irradiation, radiation-induced liver disease (RILD) is a potential complication [9, 10]. Nevertheless, due to its parallel architecture model of radiobiology, the liver can tolerate high doses to small volumes as long as the mean dose to the uninvolved hepatic tissue is kept below the threshold for severe RILD. For single doses of 1.5 Gy bidaily and α/β = 2 Gy, threshold mean liver doses of 30–32 Gy correspond to respective biologically effective doses (BEDs) of 52.5–55.1 Gy [11]. Further potential severe side-effects include gastric and bowel ulcers or perforations and chest wall fibrosis and necrosis. Overall, severe toxicities ≥ grade III can occur in up to 12 to 35% of patients undergoing liver SBRT [12, 13].

More recently, technological advances in target definition, treatment planning and methods of image guidance have enabled precise local ablative treatments of small hepatic lesions by applying SBRT. SBRT allows for safe delivery of large single doses of highly conformal radiation with steep dose gradients to the surrounding healthy tissue over a limited number of fractions. Due to its spatial precision, SBRT permits the administration of tumoricidal radiation doses to hepatic metastases, while sparing organs at risk (OARs) including the surrounding healthy hepatic tissue and hence lowering toxicity. Several retrospective and prospective series reported 1- and 3-year local control (LC) rates of 56–100% and 45–100% following SBRT for liver metastases, respectively [12, 14–18]. Similar to pulmonary SBRT, a dose–response relationship is assumed for hepatic SBRT [14, 18]. A recent meta-analysis by Ohri et al. reported significantly superior 3-year LC of 93% for hepatic metastases treated with biologically effective doses (BEDs) exceeding 100 Gy than for those irradiated with BEDs < 100 Gy with 3-year LC of only 65% [18]. However, metastases in close proximity to organs at risk (OARs) (stomach, duodenum, bowel) as well as large central metastases often cannot be treated with sufficiently high doses due to the increased risk of toxicity [19–21]. The applied maximum dose to the stomach and the bowel is known to significantly correlate with the risk of severe gastrointestinal toxicity [22]. Hence, currently only lower total doses have been applied for such lesions reducing the possibility of long-term local control.

Abdominal organs such as the liver and respective tumors are subject to movement caused by breathing and positional drifts in the body of several centimeters during treatment [23–26]. Motion management strategies are therefore crucial for a precise and safe application of high-dose liver SBRT. Clinically established strategies for motion compensation include breath hold techniques or continuous irradiation in free breathing with an internal target volume (ITV) [12, 27, 28]. The ITV concept, which is most widely used, accounts for tumor movement by incorporating tumor motion on several breathing phases assessed by a 4-dimensional (4D) CT [29]. As long as fraction times do not exceed a certain time and sufficient margins are provided, the ITV can be regarded as a robust concept for the consideration of tumor motion [30].

In routine clinical practice, low dose computed tomography scans (“cone-beam CT” (CBCT)) are generally used for daily image guidance of patient positioning, tumor location and alterations in patient anatomy (image-guided radiotherapy, IGRT). However, image-guidance for hepatic SBRT is challenged due to low tumor visibility in CBCT images and related soft tissue contrast. Consequently, for precisely targeting hepatic metastases, some centers invasively implant fiducial markers in the liver near the tumor for topographic orientation [31]. Furthermore, daily application of CBCTs is accompanied by the exposure of an additional amount of radiation dose, which in turn might theoretically even lead to an increased risk of secondary malignancies [32]. For treatment planning, magnetic resonance imaging (MRI) has gained a fundamental role in daily clinical practice, especially with regard to detecting and characterizing abdominal malignancies, such as hepatobiliary cancer and liver metastasis [33]. MRI with its superior soft tissue contrast enhances tumor delineation by enabling superior distinction between cancerous and normal tissues [34]. Additionally, functional MRI with modern, optimized sequences allows for non-invasive assessment of tissue perfusion, diffusion or cellular density, exceeding the morphological characterization of conventional MRI [35, 36].

MR-guided radiotherapy (MRgRT) has recently become clinically available, providing an excellent soft tissue contrast for precise detection of the tumor position and potential daily changes in patient anatomy without additional radiation dose [37, 38]. MR-guidance further offers the possibility to visualize tumor volume and nearby OARs during the whole treatment session (cine MRI). Safety margins and hence the irradiated volume can be decreased for hepatic MR-guided SBRT, thereby reducing the risk of potential toxicity [39, 40]. Hepatic SBRT of smaller target volumes might offer the possibility of dose escalation for increasing local control.

MRgRT further allows for online plan adaptation in response to specific changes in tumor and OAR anatomy that may occur during the course of treatment. In conventional radiotherapy, one treatment plan is generated based on the patient anatomy during simulation CT imaging. However, significant organ motion is known to occur between different treatment fractions, e.g. due to varying filling of hollow organs or tumor shrinkage in response to therapy (interfractional organ motion) [41, 42]. MRgRT enables daily imaging of sufficient quality to permit immediate plan adjustments in response to the anatomy of the day, while the patient keeps lying on the treatment couch [37, 43–45]. Online plan adaptation allows for superior sparing of OARs and offers the possibility for dose escalation hereby potentially improving local control rates [40, 44]. Particularly for hepatic metastases located near the liver margin, superior visualization before and during irradiation allows a safe treatment with high doses near organs such as bowel or stomach, where positional uncertainties could result in a dose that exceeds OAR tolerance [46]. With real-time imaging, treatment plans for hepatic metastases can be adapted as needed and RT doses may be better personalized.

Consequently, oncologic treatments with hybrid MRI-Linear accelerators might improve treatment outcome both with regard to tumor response and treatment related side effects, as for the possibility of monitoring treatment related changes by different morphologic and functional MRI sequences.

Up to now, studies for MRgRT of hepatic lesions are scarce with mainly retrospective analyses, observational studies and small case series, limited to a maximum of 21 patients with liver metastases per study [39, 40, 46–52]. However, MRgRT is very staff-intense and time-consuming compared to standard CT-guided hepatic SBRT [53, 54]. Hence, prospective studies are needed to assess which patients profit most from this new technique. The aim of the present study is therefore to evaluate potential benefits of MRgRT compared to ITV-based SBRT as one of the current, and probably the most widely-used, state-of-the-art standard techniques. Non-inferiority of MRgRT compared to standard ITV-based SBRT for hepatic metastases will be evaluated in respect to gastrointestinal and hepatobiliary toxicity ≥ grade III. Special attention will be paid to whether MRgRT offers a potential for dose escalation in case of critical proximity of hepatic metastases to gastrointestinal OARs, currently limiting application of a sufficient BED of ≥ 100 Gy in certain cases.

## Methods/design

### Trial aim

The MAESTRO trial compares online adaptive MR-guided SBRT with state-of-the-art ITV-based SBRT of hepatic metastases to strive for prospective, randomized evidence regarding toxicity as well as potential dosimetric and clinical advantages of oMRgRT for dose escalation in critically located hepatic metastases adjacent to radiosensitive OARs.

### Trial design

**Fig. 1:**
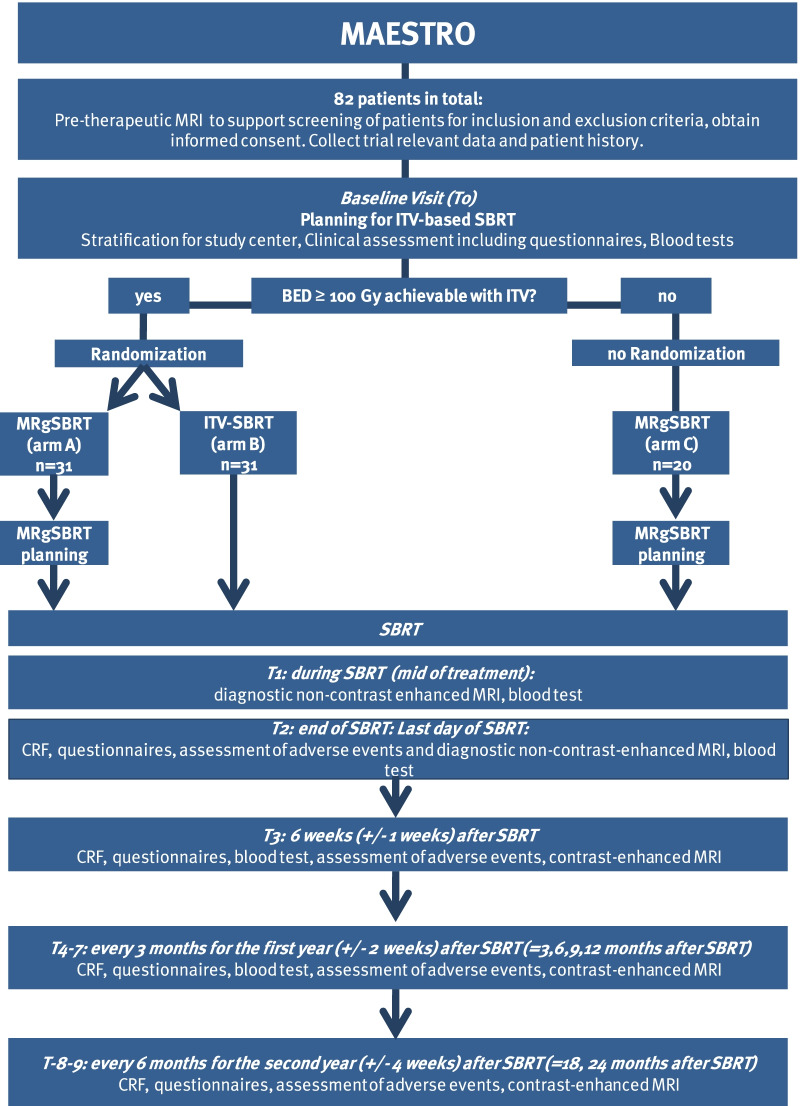
Trial flow-chart

The MAESTRO trial is a multi-center, prospective three-armed phase II study. It has been designed by the study initiators at the Department of Radiation Oncology of the University of Heidelberg. The trial is performed at the University of Heidelberg, Department of Radiation Oncology and at University Hospital, LMU Munich, Department of Radiation Oncology. Further study sites might be included. A list of study sites can be obtained from the corresponding author on request. The University of Heidelberg is responsible for the coordination and trial management, as well as quality assurance including reporting, monitoring and database management. The current version of the study protocol is version 1.4 from November, 19th 2021 (Additional file 1). The study workflow and treatment arms are depicted in Fig. 1. At least 82 patients fulfilling the inclusion criteria will be enrolled.

### Inclusion criteria

Patients with hepatic metastases of a solid malignant non-hepatic primary tumor (no hepatocellular or cholangiocellular carcinoma, no germ cell tumor, leukemia or lymphoma) meeting all of the following criteria are considered for admission to the trial:

- 1–3 Active hepatic metastases confirmed by pre-therapeutic MRI
- Indication for SBRT of 1–3 hepatic metastases
- Maximum diameter of each hepatic metastasis ≤ 5 cm (in case of 3 metastases: sum of diameters ≤ 12 cm)
- Age ≥ 18 years of age
- Karnofsky Performance Score ≥ 60% / ECOG grade 0–2
- Ability to lie still on the radiotherapy treatment couch for at least one hour
- Ability to hold one’s breath for more than 25 s
- For women with childbearing potential, adequate contraception
- Ability of subject to understand character and individual consequences of the clinical trial
- Written informed consent (must be available before enrolment in the trial).

### Exclusion criteria

Patients presenting with any of the following criteria will not be included in the trial:

- Patients after liver transplantation
- Impairment of liver function to an extent contraindicating radiotherapy (to the discretion of the treating radiation oncologist)
- Active acute hepatic/biliary infection (e.g. hepatitis, cholangitis, cholecystitis)
- Previous radiotherapy of the hepatobiliary system, if previous and current target volumes overlap
- Patients who have not yet recovered from acute toxicities of prior therapies
- Claustrophobia
- Pregnant or lactating women

Contraindications against performing contrast-enhanced MRI scans (pacemakers or other implants making MRI impossible, allergy to gadolinium-based contrast agent)

Participation in another competing clinical study or observation period of competing trials

Concomitant systemic therapy or other anti-tumor medication are not part of the study treatment and are only allowed after consultation with the treating radiation oncologist.

All patients receive a pre-treatment hepatic MRI for diagnostic and treatment planning purposes.

### Randomization

Patients are randomized according to initial treatment planning. If a BED ≥ 100 Gy can be achieved with standard ITV-based planning, patients are randomized to MRgRT (arm A) or ITV-SBRT (arm B) in a 1:1 fashion. If a BED ≥ 100 Gy (α/β = 10 Gy) cannot be achieved with ITV-SBRT planning, the patient is treated with the highest possible dose using MRgRT (arm C). To achieve comparable intervention groups (arms A and B), patients are allocated in a concealed fashion in a 1:1 ratio by means of randomization using a centralized web-based tool (www.randomizer.at). Randomization is stratified with respect to the factor center. Block randomization with varying block lengths will be performed to achieve in total equal group sizes for randomization.

### Treatment planning and dose prescription

Patient eligibility for SBRT is tested before enrollment in the trial. Training aims to enhance the ability to follow respiratory instructions including free breathing and deep inspiration breath-hold.

In a first step, treatment planning is performed with contrast-enhanced and non-enhanced CT scans in free breathing in a supine position. Furthermore, patients undergo a 4D-CT with typically 8 phases to assess tumor motion in free-breathing. Patients are immobilized according to standard practice in the specific center. Based on pre-therapeutic MRI and planning CT, SBRT is planned using an internal target volume (ITV) concept. Target volume delineation for ITV-based SBRT is performed as follows:

- Gross tumor volume (GTV): macroscopic, contrast-enhanced tumor in the planning MRI and, if definable, in the planning CT
- Internal Target Volume (ITV): sum of all GTV contours derived from different breathing phases assessed via 4D-CT
- Clinical Target Volume (CTV): ITV + 5 mm
- Planning Target Volume (PTV): CTV + at least 5 mm, depending on the applied image-guidance (e.g. CBCT)

Dose is prescribed to the surrounding 65–95% isodose (preferably: 65–80% isodose) in up to 10 fractions. For hepatic metastases, α/β = 10 Gy is assumed. Dose constraints for OARs are summarized in Table 1. These dose constraints resemble UK Consensus on Normal Tissue Dose Constraints for Stereotactic Radiotherapy [55]. Further dose constraints of normal tissue are respected according to German and international guidelines [12, 55, 56]. If a BED of ≥ 100 Gy can be achieved with an ITV-concept, patients are randomized to arms A or B. If a BED of ≥ 100 Gy is not achievable using an ITV approach, patients are treated with MRgRT in arm C without randomization.

**Table 1:** Dose constraints for organs at risk depending on fractionation

Organ at risk	Dose constraints (Gy)				
Number of fractions	3 Fractions	5 Fractions	6 Fractions	8 Fractions	10 Fractions
Uninvolved liver (= liver-CTV) ≥ 700 cm^3^	< 19.2	< 24	< 26	< 29	< 32
Stomach D_0.5cm3_	< 22.2	< 35	< 37	< 40	< 43.5
Duodenum D_0.5cm3_	< 22.2	< 35	< 37	< 40	< 43.5
Bowel D_0.5cm3_	< 25.2	< 35	< 37	< 40	< 43.5
Sigma/Rectum D_0.5cm3_	< 28.2	< 34	< 37	< 41	< 44.0
Esophagus D_0.5cm3_	< 25.2	< 34	< 36	< 40	< 43.5
Heart D_0.5cm3_	< 25.0	< 29.0	< 31.5	< 60.0	< 66.0
Kidneys Mean dose	< 8.5	< 10.0	< 10.8	< 11.5	< 12.0
Spinal cord D_0.1cm3_	< 21.6	< 27.0	< 29.0	< 32.0	< 35.0

CTV, clinical target volume; D_xcm3_, dose which is received by less than × cm^3^

In a second step, patients to be treated with MRgRT receive a simulation at the MR-Linac for MRgRT planning. If necessary, they also undergo a specific planning CT in deep-inspiration breath-hold (non-contrast-enhanced). In the MRgRT arms, patients are immobilized in supine position, and fitted with the MRI receiver coils.

For MRgRT (arms A and C), target volume delineation is performed as follows:

- Gross tumor volume (GTV): macroscopic, contrast-enhanced tumor in the planning MR and, if definable, in the planning CT
- Clinical Target Volume (CTV): GTV + 5 mm
- Planning Target Volume (PTV): CTV + 3 mm

Dose prescription and constraints for MRgRT are the same as for ITV-based SBRT as described above. In arm A, a BED of ≥ 100 Gy must be prescribed. In arm C, patients are treated via MRgRT with the highest achievable dose as deemed by the treating radiation oncologist.

For MRgRT (arms A and C), daily OAR contour adaption is performed within the region PTV_expand_ slightly adapted from the recommendations by Bohoudi et al. [44]. The PTV_expand_ is the PTV enlarged by 3 cm in medio-lateral and anterior–posterior direction as well as 1 cm in the cranio-caudal direction. The need to adapt the treatment plan is decided upon by the treating physician based on dose prediction. For MRgRT, real-time cine MR imaging is used for respiratory gating. Gating thresholds are defined by the treating physician.

### Trial objectives

The primary endpoint is defined as the occurrence of treatment-related gastrointestinal or hepatobiliary toxicity of grade III or higher according to Common Terminology Criteria for Adverse Events (CTCAE) V5.0 assessed within the first year after SBRT. The primary aim of the study is the assessment of non-inferiority of MRgRT to ITV-SBRT regarding the primary endpoint, assuming a non-inferiority margin of $\delta =10\%.$ Toxicity of CTCAE III° or higher is generally low, between below 1% [57] and 7.7% [46] according to published literature. Therefore, a non-inferiority-margin of $\updelta$=10% is deemed adequate.

Secondary objectives include further toxicity according to CTCAE V5.0, quality of life according to EORTC QLQ C-30 and EORTC QLQ LMC-21, local control (treated lesions), locoregional control (whole liver), distant tumor control, progression-free survival (PFS) and overall survival (OS).

QoL will be measured with the help of the validated 30-item self-assessment questionnaire of the European Organization for Research and Treatment of Cancer (EORTC QLQ-C30, version 3.0). It is composed of five multi-item functional scales (physical, role, emotional, cognitive, and social function), three multi-item symptom scales (fatigue, pain, nausea/vomiting) combined with a global health and quality-of life scale. The other six single items assess further symptoms (dyspnea, insomnia, appetite loss, constipation and diarrhea) that are often reported by cancer patients as well as financial difficulties [58]. The QLQ-C30 questionnaire will be complemented with the colorectal liver cancer module of the EORTC (QLQ-LMC-21), initially designed for patients with colorectal liver metastases [59, 60]. QLQ-LMC-21 comprises another 21 items in five scales (nutritional, fatigue, pain, emotional) and six single items assessing further symptoms (weight, loss of taste, xerostomia, oral mucositis, paresthesia, jaundice).

For patients in the MRgRT arms, an in-house developed patient-reported questionnaire will be applied after the first fraction of radiotherapy and at the end of radiotherapy to assess acceptance of MR-guided radiotherapy [53]. The questionnaire consists of questions regarding potential MR-related experiences and complaints (e.g. noise, bore size, fixation with coils). Furthermore, the perception of their active role during gated dose delivery SBRT is assessed. Items are scored using a 5-point scale.

MR-guided and ITV-based SBRT will be compared with respect to toxicity and local control, treatment plan and irradiation parameters including potential BED increase and OAR dose decrease using MRgRT as well as required adaption frequency and dosimetric benefit of adaption for MRgRT. Morphological and functional changes in MRI during and after radiotherapy will be evaluated, in particular with regard to dose distribution [61, 62]. Potential prognostic biomarkers such as plasma levels of hepatic growth factor (HGF) and interleukines (e.g. IL-6 and IL-8) will also be assessed [63].

### Study visits and follow-up

After screening of patients for inclusion and exclusion criteria, successful completion of breath hold assessment as well as having received informed consent, appropriate patients will be recruited to the trial.

Study relevant data will be collected and patient history will be assessed, including demographic data, medical history, physical examination, comorbidities and concomitant medication, laboratory evaluation including blood count, liver function parameters and potential biomarkers.

The baseline visit (T0) will be scheduled after trial inclusion and prior to the first fraction of SBRT. During the baseline visit (T0) a clinical assessment, as well as analysis of quality of life (using EORTC QLQ C-30 and EORTC QLQ LMC-21) is planned. Baseline symptoms and toxicities will be assessed according to CTCAE V5.0.

Further evaluations will be scheduled during SBRT (mid of treatment, T1), at the last day of treatment (T2), 6 weeks after radiotherapy (T3), every 3 months after radiotherapy during the first year (T4-7) and then every 6 months up to completion of a follow-up of at least 2 years (T8, T9). Study visits are also depicted in the flowchart and in Table 2.

**Table 2:** Screening, treatment and follow-up visits

	Screening	Baseline (before SBRT)	Mid of SBRT	End of SBRT	Follow-up		
					6 weeks after SBRT	1st year after SBRT: every 3 months	2nd year after SBRT: every 6 months
		T0	T1	T2	T3	T4-7	T8-9
Medical history		x		x	x	x	x
EORTC QLQ-C30 and LMC21		x		x	x	x	x
MR-Linac questionnaire (only treatment arm A)		x		x			
Documentation of medication		x		x	x	x	x
Documentation of Adverse Events		x		x	x	x	x
Case report form		x		x	x	x	x
Blood test		x	x	x	x	x	
Breathhold assessment (> 25 s?)	x						
MRI (c = contrast enhanced, n = without)	x (c)		x (n)	x (n)	x (c)	x (c)	x (c)

### Statistical analysis

The sample size calculation is based on the primary comparison of the rates of gastrointestinal and hepatobiliary toxicity CTCAE of grade III or higher between the two treatment groups (arm A and B). A total of 62 patients are needed to assess non-inferiority by means of a Farrington-Manning test of MRgRT to ITV-SBRT with a power of 80% at a one-sided significance level of 10%, with allowance for 5% loss to follow-up and with the use of a clinically relevant non-inferiority margin of $\delta$ =10%, when assuming toxicity rates of pMRgRT = 2% and pITV-SBRT = 5%. It is expected that using a shifted Mantel–Haenszel type test for non-inferiority adjusting for the factor centre will yield an increased power.

To achieve a comparable arm C (BED < 100 Gy with ITV-SBRT) for the secondary objectives, the study will recruit patients until at least 20 analyzable patients are present in arm C. Due to the fact that patients in arm C are still treated with the best possible treatment, this approach is ethically justifiable.

Sample size calculation was performed using PASS 16.0.3.

The primary analysis will be based on the full analysis set (FAS) including all enrolled patients according to the intention to treat principle. The hypotheses for the primary analysis are

$${H}_{0}:{p}_{\mathrm{MRgRT}}- {p}_{\mathrm{ITV}-\mathrm{SBRT }}\ge \delta vs. {H}_{1}:{p}_{\mathrm{MRgRT }}- {p}_{\mathrm{IITV}-\mathrm{SBRT }}<\delta$$

($\delta$ =10% non-inferiority margin), where ${p}_{\mathrm{MRgRT}}$ and ${p}_{\mathrm{ITV}-\mathrm{SBRT}}$ are the probabilities for an occurrence of a gastrointestinal and hepatobiliary toxicity CTCAE of grade III or higher for the MRgRT (arm A) and the ITV-SBRT (arm B) group, respectively. Non-inferiority of MRgRT as compared to ITV-SBRT will be tested at a one-sided significance level of α = 0.1 using a Mantel–Haenszel type test for non-inferiority adjusting for the stratum “centre”. Missing values for the primary outcome will be imputed using multiple imputation. Sensitivity analyses will be performed by means of conducting an analysis for the per-protocol (PP) population (based on those patients without major protocol violation).

Analysis of the secondary endpoints will also be based on the FAS. In order to compare all three treatment groups with regard to the primary endpoint, a (descriptive) Chi-squared test will be used. The secondary endpoints distant tumor control, overall survival and progression-free survival will be analyzed using Kaplan–Meier-Curves. The 1-year and 2-year survival rates as well as the median survival rate will be provided alongside two-sided 95%-confidence intervals. Descriptive pairwise log-rank tests stratified for “centre” will be conducted to compare all three treatment groups. The secondary endpoints local and locoregional control will be analyzed via cumulative incidence functions, taking the competing event death into account.

The other secondary endpoints and the patients’ characteristics will be displayed by descriptive measures. Descriptive pairwise comparisons between all three treatment groups will be performed. Continuous variables will be described using number of non-missing values, mean, standard deviation, median, Q1, Q3, minimum and maximum. For binary or categorical variables absolute and relative frequencies will be provided. Furthermore, two-sided 95%-confidence intervals will be calculated.

The safety analysis is based on the safety set including all patients who received one of the study treatments, and includes calculation of frequencies and rates of adverse and serious adverse events together with corresponding 95%-confidence intervals.

Further details of the analysis will be specified in the statistical analysis plan (SAP) which will be finalized before database closure. All analyses will be done using SAS version 9.4 or higher.

## Discussion

SBRT of hepatic metastases can achieve excellent long-term local control rates if a sufficient BED is achieved [18, 57]. In general, a BED exceeding 100 Gy should be aspired. Following technological innovations, prescribed BED to hepatic metastasis has increased over the last decades [57]. However, application of a sufficient BED is still limited in metastases close to radiosensitive OAR as well as in large central metastases [19–21]. MRgRT with its technical innovations of real-time imaging, gating and online on-table adaption is believed to improve hepatic SBRT. First publications on MR-guided SBRT of liver lesions show promising results [39, 40, 46–52]. With a maximum of 21 patients with liver metastases per study and no randomized controlled trials (RCT), high-level evidence is still missing.

However, high-level evidence is needed to justify the prolonged treatment duration and extensive expenses related to MRgRT [64, 65]. As long-term clinical evidence is lacking, such is data on the economic impact of MRgRT on health systems. For MRgRT of prostate cancer, a modelled analysis predicted cost-effectiveness compared to normofractionated radiotherapy. But compared to other hypofractionated irradiation techniques, MRgRT could only be cost-effective in the case of significant reduction of adverse events [66]. This might also apply to liver SBRT. In a time-driven activity-based costing comparison of 5-fraction SBRT of hepatocellular carcinoma using either a CBCT-equipped linac and implanted fiducial markers or a MR-Linac, MRgRT costs were estimated to be 18% higher compared to CBCT-based SBRT ($8622 vs. $7306). Notably, the addition of adaptive treatments (which is one of the main drivers of increased efforts and costs in MRgRT) further increased the costs by $529 per adapted fraction [67]. With all of this considered, MRgRT can be estimated to be more expensive than ITV-based SBRT. Therefore, it is important to identify patients who benefit most from MRgRT.

A search of the ClinicalTrials.gov database revealed five other recruiting studies on adaptive MRgRT of hepatic metastases:

- NCT04115254, an umbrella protocol for a phase I-II study on adaptive MRgRT of various disease sites, including the liver. The trial will assess feasibility, safety and efficacy of MRgRT without an intrinsic control group [68].
- NCT04682847: In this single-arm prospective observation study, 25 patients with primary and metastatic hepatic malignancies and hepatic cirrhosis will receive Iron Oxide Nanoparticles (SPION) Cellular Magnetic Resonance Imaging in combination with MRgRT [69].
- NCT04242342: In this single group assignment study, 46 patients with primary and secondary liver tumors will undergo MRgRT. Depending on distance to OAR, prescribed dose will be 5 × 10 Gy or 6 × 10 Gy [70].
- NCT03582189: This is a single arm, prospective feasibility study in 30 patients (10 of which with liver metastases), evaluating an off-line MR approach with diagnostic MR imaging before every fraction and potential adaption [71].
- NCT04020276: In this single-group assignment study, 48 patients with liver metastasis of any primary will be treated in 5 fractions to a safe maximum tolerated dose (maximum dose 80 Gy in five fractions respecting predefined dose constraints), with dose limiting toxicity defined as grade 3 or higher. An additional confirmatory expansion cohort of patients with liver metastases from colorectal cancer only will be recruited afterwards [72].

Based on the present clinical evidence for liver SBRT, the main factor for compromised local control seems to be an impaired BED. Given that local control is favorable in most cases for a BED > 100 Gy, we believe that in metastases far from radiosensitive OAR, the clinically more relevant benefit of MRgRT might be a reduction of side effects instead of a further dose escalation wherever possible. In fact, radiomic approaches during SBRT might even allow for identification of well-responding metastases and consecutive dose de-escalation in the future [64].

For critically localized metastases close to sensitive OAR, dose escalation while simultaneously keeping OAR dose and thus toxicity low is of course the main potential benefit of MRgRT.

In a first step, the present study aims at demonstrating safety of MRgRT by proving non-inferiority to standard ITV-based SBRT with regard to gastrointestinal and hepatobiliary toxicity ≥ grade III. In a second step, we plan to demonstrate the possibility of isotoxic dose escalation in critically located hepatic metastases (study arm C), where a sufficient BED would not be achievable with state-of-the-art standard ITV-based SBRT.

None of the previously published work or currently recruiting trials on oMRgRT is a randomized controlled trial. As a comparison of randomized controlled trials in oncology with observational paired analyses showed, the latter failed to reproduce or predict RCT results better than by mere chance [73]. Therefore, we opted for a randomized controlled study design.

The MAESTRO trial aims to provide higher-level evidence for the potential clinical benefit of MR-guided, online adaptive SBRT for dose escalation in critically located hepatic metastases adjacent to radiosensitive OARs.

## Supplementary Information

**Additional file 1.** Study protocol.

## Data Availability

The data is collected, managed and processed electronically in the in-house research database of the primary study center. To ensure data quality and consistency, internal quality control measures will be carried out. The originals of all central study documents are kept at the study center for at least 10 years after the final report has been prepared. The dataset used and analyzed during the current study will be available from the corresponding author on reasonable request. Regulatory authorities may request access to all source documents, case report forms and other trial documentation.

## Abbreviations

BED: Biologically Effective Dose
CBCT: Cone-Beam Computed Tomography
CT: Computed Tomography
CTCAE: Common Terminology Criteria for Adverse Events
CTV: Clinical Target Volume
FAS: Full Analysis Set
GTV: Gross Tumor Volume
Gy: Gray
IGRT: Image-guided Radiotherapy
IMRT: Intensity-modulated Radiotherapy
ITV: Internal Target Volume
ITV-SBRT: ITV-based Stereotactic Body Radiotherapy
MR(I): Magnetic Resonance (Imaging)
(o)MRgRT: (Online) Magnetic Resonance-guided Radiotherapy
OAR: Organ at Risk
PP: Per-protocol
PTV: Planning Target Volume
RCT: Randomized Controlled Trial
RILD: Radiation-induced Liver Disease
RT: Radiotherapy
SBRT: Stereotactic Body Radiotherapy

## References

[1] Adam R, Chiche L, Aloia T, Elias D, Salmon R, Rivoire M (2006). Hepatic resection for noncolorectal nonendocrine liver metastases: analysis of 1,452 patients and development of a prognostic model. Ann Surg.

[2] House MG, Ito H, Gonen M, Fong Y, Allen PJ, DeMatteo RP (2010). Survival after hepatic resection for metastatic colorectal cancer: trends in outcomes for 1,600 patients during two decades at a single institution. J Am Coll Surg.

[3] Nordlinger B, Sorbye H, Glimelius B, Poston GJ, Schlag PM, Rougier P (2013). Perioperative FOLFOX4 chemotherapy and surgery versus surgery alone for resectable liver metastases from colorectal cancer (EORTC 40983): long-term results of a randomised, controlled, phase 3 trial. Lancet Oncol.

[4] Tomlinson JS, Jarnagin WR, DeMatteo RP, Fong Y, Kornprat P, Gonen M (2007). Actual 10-year survival after resection of colorectal liver metastases defines cure. J Clin Oncol: Off J Am Soc Clin Oncol.

[5] Smith JJ, D'Angelica MI (2015). Surgical management of hepatic metastases of colorectal cancer. Hematol Oncol Clin North Am.

[6] Cummings LC, Payes JD, Cooper GS (2007). Survival after hepatic resection in metastatic colorectal cancer: a population-based study. Cancer.

[7] Ruers T, Van Coevorden F, Punt CJ, Pierie JE, Borel-Rinkes I, Ledermann JA (2017). Local treatment of unresectable colorectal liver metastases: results of a randomized phase II trial. J Natl Cancer Inst.

[8] Bretschneider T, Ricke J, Gebauer B, Streitparth F (2016). Image-guided high-dose-rate brachytherapy of malignancies in various inner organs—technique, indications, and perspectives. J Contemp Brachyther.

[9] Munoz-Schuffenegger P, Ng S, Dawson LA (2017). Radiation-induced liver toxicity. Semin Radiat Oncol.

[10] Pan CC, Kavanagh BD, Dawson LA, Li XA, Das SK, Miften M (2010). Radiation-associated liver injury. Int J Radiat Oncol Biol Phys.

[11] Dawson LA, Normolle D, Balter JM, McGinn CJ, Lawrence TS, Ten Haken RK (2002). Analysis of radiation-induced liver disease using the Lyman NTCP model. Int J Radiat Oncol Biol Phys.

[12] Sterzing F, Brunner TB, Ernst I, Baus WW, Greve B, Herfarth K (2014). Stereotactic body radiotherapy for liver tumors: principles and practical guidelines of the DEGRO Working Group on Stereotactic Radiotherapy. Strahlenther Onkol.

[13] Bae SH, Kim MS, Cho CK, Kang JK, Lee SY, Lee KN (2012). Predictor of severe gastroduodenal toxicity after stereotactic body radiotherapy for abdominopelvic malignancies. Int J Radiat Oncol Biol Phys.

[14] Chang DT, Swaminath A, Kozak M, Weintraub J, Koong AC, Kim J (2011). Stereotactic body radiotherapy for colorectal liver metastases: a pooled analysis. Cancer.

[15] Rusthoven KE, Kavanagh BD, Cardenes H, Stieber VW, Burri SH, Feigenberg SJ (2009). Multi-institutional phase I/II trial of stereotactic body radiation therapy for liver metastases. J Clin Oncol: Off J Am Soc Clin Oncol.

[16] Yamashita H, Onishi H, Matsumoto Y, Murakami N, Matsuo Y, Nomiya T (2014). Local effect of stereotactic body radiotherapy for primary and metastatic liver tumors in 130 Japanese patients. Radiat Oncol.

[17] Doi H, Beppu N, Kitajima K, Kuribayashi K (2018). Stereotactic body radiation therapy for liver tumors: current status and perspectives. Anticancer Res.

[18] Ohri N, Tome WA, Mendez Romero A, Miften M, Ten Haken RK, Dawson LA (2018). Local control after stereotactic body radiation therapy for liver tumors. Int J Radiat Oncol Biol Phys.

[19] Doi H, Shiomi H, Masai N, Tatsumi D, Igura T, Imai Y (2016). Threshold doses and prediction of visually apparent liver dysfunction after stereotactic body radiation therapy in cirrhotic and normal livers using magnetic resonance imaging. J Radiat Res.

[20] Kavanagh BD, Pan CC, Dawson LA, Das SK, Li XA, Ten Haken RK (2010). Radiation dose-volume effects in the stomach and small bowel. Int J Radiat Oncol Biol Phys.

[21] Miften M, Vinogradskiy Y, Moiseenko V, Grimm J, Yorke E, Jackson A (2018). Radiation dose-volume effects for liver SBRT. Int J Radiat Oncol Biol Phys.

[22] Bae SH, Kim M-S, Cho CK, Kang J-K, Lee SY, Lee K-N (2012). Predictor of severe gastroduodenal toxicity after stereotactic body radiotherapy for abdominopelvic malignancies. Int J Radiat Oncol Biol Phys.

[23] Bertholet J, Worm ES, Fledelius W, Hoyer M, Poulsen PR (2016). Time-resolved intrafraction target translations and rotations during stereotactic liver radiation therapy: implications for marker-based localization accuracy. Int J Radiat Oncol Biol Phys.

[24] Poulsen PR, Worm ES, Petersen JB, Grau C, Fledelius W, Hoyer M (2014). Kilovoltage intrafraction motion monitoring and target dose reconstruction for stereotactic volumetric modulated arc therapy of tumors in the liver. Radiother Oncol: J Eur Soc Ther Radiol Oncol.

[25] Worm ES, Hoyer M, Fledelius W, Hansen AT, Poulsen PR (2013). Variations in magnitude and directionality of respiratory target motion throughout full treatment courses of stereotactic body radiotherapy for tumors in the liver. Acta Oncol.

[26] Dhont J, Vandemeulebroucke J, Burghelea M, Poels K, Depuydt T, Van Den Begin R (2018). The long- and short-term variability of breathing induced tumor motion in lung and liver over the course of a radiotherapy treatment. Radiother Oncol: J Eur Soc Ther Radiol Oncol.

[27] Naumann P, Batista V, Farnia B, Fischer J, Liermann J, Tonndorf-Martini E (2020). Feasibility of optical surface-guidance for position verification and monitoring of stereotactic body radiotherapy in deep-inspiration breath-hold. Front Oncol.

[28] Cusumano D, Dhont J, Boldrini L, Chiloiro G, Teodoli S, Massaccesi M (2018). Predicting tumour motion during the whole radiotherapy treatment: a systematic approach for thoracic and abdominal lesions based on real time MR. Radiother Oncol: J Eur Soc Ther Radiol Oncol.

[29] Hof H, Rhein B, Haering P, Kopp-Schneider A, Debus J, Herfarth K (2009). 4D-CT-based target volume definition in stereotactic radiotherapy of lung tumours: comparison with a conventional technique using individual margins. Radiother Oncol.

[30] Cusumano D, Dhont J, Boldrini L, Chiloiro G, Romano A, Votta C (2020). Reliability of ITV approach to varying treatment fraction time: a retrospective analysis based on 2D cine MR images. Radiat Oncol.

[31] Worm ES, Bertholet J, Høyer M, Fledelius W, Hansen AT, Larsen LP (2016). Fiducial marker guided stereotactic liver radiotherapy: is a time delay between marker implantation and planning CT needed?. Radiother Oncol.

[32] Kan MW, Leung LH, Wong W, Lam N (2008). Radiation dose from cone beam computed tomography for image-guided radiation therapy. Int J Radiat Oncol Biol Phys.

[33] Lincke T, Zech CJ (2017). Liver metastases: detection and staging. Eur J Radiol.

[34] Khoo VS, Joon DL (2006). New developments in MRI for target volume delineation in radiotherapy. Br J Radiol.

[35] Bonekamp D, Deike K, Wiestler B, Wick W, Bendszus M, Radbruch A (2015). Association of overall survival in patients with newly diagnosed glioblastoma with contrast-enhanced perfusion MRI: comparison of intraindividually matched T1 - and T2 (*)-based bolus techniques. J Magn Reson Imaging.

[36] Donaldson SB, Betts G, Bonington SC, Homer JJ, Slevin NJ, Kershaw LE (2011). Perfusion estimated with rapid dynamic contrast-enhanced magnetic resonance imaging correlates inversely with vascular endothelial growth factor expression and pimonidazole staining in head-and-neck cancer: a pilot study. Int J Radiat Oncol Biol Phys.

[37] Acharya S, Fischer-Valuck BW, Kashani R, Parikh P, Yang D, Zhao T (2016). Online magnetic resonance image guided adaptive radiation therapy: first clinical applications. Int J Radiat Oncol Biol Phys.

[38] Noel CE, Parikh PJ, Spencer CR, Green OL, Hu Y, Mutic S (2015). Comparison of onboard low-field magnetic resonance imaging versus onboard computed tomography for anatomy visualization in radiotherapy. Acta Oncol.

[39] Boldrini L, Cellini F, Manfrida S, Chiloiro G, Teodoli S, Cusumano D (2018). Use of indirect target gating in magnetic resonance-guided liver stereotactic body radiotherapy: case report of an oligometastatic patient. Cureus.

[40] Henke L, Kashani R, Robinson C, Curcuru A, DeWees T, Bradley J (2018). Phase I trial of stereotactic MR-guided online adaptive radiation therapy (SMART) for the treatment of oligometastatic or unresectable primary malignancies of the abdomen. Radiother Oncol: J Eur Soc Ther Radiol Oncol.

[41] Li XA, Qi XS, Pitterle M, Kalakota K, Mueller K, Erickson BA (2007). Interfractional variations in patient setup and anatomic change assessed by daily computed tomography. Int J Radiat Oncol Biol Phys.

[42] Wysocka B, Kassam Z, Lockwood G, Brierley J, Dawson LA, Buckley CA (2010). Interfraction and respiratory organ motion during conformal radiotherapy in gastric cancer. Int J Radiat Oncol Biol Phys.

[43] Henke LE, Olsen JR, Contreras JA, Curcuru A, DeWees TA, Green OL (2019). Stereotactic MR-guided online adaptive radiation therapy (SMART) for ultracentral thorax malignancies: results of a phase 1 trial. Adv Radiat Oncol.

[44] Bohoudi O, Bruynzeel AME, Senan S, Cuijpers JP, Slotman BJ, Lagerwaard FJ (2017). Fast and robust online adaptive planning in stereotactic MR-guided adaptive radiation therapy (SMART) for pancreatic cancer. Radiother Oncol.

[45] Henke LE, Contreras JA, Green OL, Cai B, Kim H, Roach MC (2018). Magnetic resonance image-guided radiotherapy (MRIgRT): a 4.5-year clinical experience. Clin Oncol.

[46] Rosenberg SA, Henke LE, Shaverdian N, Mittauer K, Wojcieszynski AP, Hullett CR (2019). A multi-institutional experience of MR-guided liver stereotactic body radiation therapy. Adv Radiat Oncol.

[47] Feldman AM, Modh A, Glide-Hurst C, Chetty IJ, Movsas B (2019). Real-time magnetic resonance-guided liver stereotactic body radiation therapy: an institutional report using a magnetic resonance-Linac system. Cureus.

[48] Hall WA, Straza MW, Chen X, Mickevicius N, Erickson B, Schultz C (2020). Initial clinical experience of Stereotactic Body Radiation Therapy (SBRT) for liver metastases, primary liver malignancy, and pancreatic cancer with 4D-MRI based online adaptation and real-time MRI monitoring using a 1.5 Tesla MR-Linac. PLoS ONE.

[49] Weykamp F, Hoegen P, Kluter S, Spindeldreier CK, Konig L, Seidensaal K (2021). Magnetic resonance-guided stereotactic body radiotherapy of liver tumors: initial clinical experience and patient-reported outcomes. Front Oncol.

[50] Gani C, Boeke S, McNair H, Ehlers J, Nachbar M, Monnich D (2021). Marker-less online MR-guided stereotactic body radiotherapy of liver metastases at a 1.5 T MR-Linac—feasibility, workflow data and patient acceptance. Clin Transl Radiat Oncol.

[51] Rogowski P, von Bestenbostel R, Walter F, Straub K, Nierer L, Kurz C (2021). Feasibility and early clinical experience of online adaptive MR-guided radiotherapy of liver tumors. Cancers (Basel).

[52] Ugurluer G, Mustafayev TZ, Gungor G, Atalar B, Abacioglu U, Sengoz M (2021). Stereotactic MR-guided online adaptive radiation therapy (SMART) for the treatment of liver metastases in oligometastatic patients: initial clinical experience. Radiat Oncol J.

[53] Klüter S, Katayama S, Spindeldreier CK, Koerber SA, Major G, Alber M (2020). First prospective clinical evaluation of feasibility and patient acceptance of magnetic resonance-guided radiotherapy in Germany. Strahlenther Onkol.

[54] Tetar S, Bruynzeel A, Bakker R, Jeulink M, Slotman BJ, Oei S (2018). Patient-reported outcome measurements on the tolerance of magnetic resonance imaging-guided radiation therapy. Cureus.

[55] Hanna GG, Murray L, Patel R, Jain S, Aitken KL, Franks KN (2018). UK consensus on normal tissue dose constraints for stereotactic radiotherapy. Clin Oncol.

[56] Guckenberger M, Andratschke N, Alheit H, Holy R, Moustakis C, Nestle U (2014). Definition of stereotactic body radiotherapy: principles and practice for the treatment of stage I non-small cell lung cancer. Strahlenther Onkol.

[57] Andratschke N, Alheid H, Allgäuer M, Becker G, Blanck O, Boda-Heggemann J (2018). The SBRT database initiative of the German Society for Radiation Oncology (DEGRO): patterns of care and outcome analysis of stereotactic body radiotherapy (SBRT) for liver oligometastases in 474 patients with 623 metastases. BMC Cancer.

[58] Aaronson NK, Ahmedzai S, Bergman B, Bullinger M, Cull A, Duez NJ (1993). The European Organization for Research and Treatment of Cancer QLQ-C30: a quality-of-life instrument for use in international clinical trials in oncology. J Natl Cancer Inst.

[59] Kavadas V, Blazeby JM, Conroy T, Sezer O, Holzner B, Koller M (2003). Development of an EORTC disease-specific quality of life questionnaire for use in patients with liver metastases from colorectal cancer. Eur J Cancer (Oxf, Engl: 1990).

[60] Blazeby JM, Fayers P, Conroy T, Sezer O, Ramage J, Rees M (2009). Validation of the European Organization for Research and Treatment of Cancer QLQ-LMC21 questionnaire for assessment of patient-reported outcomes during treatment of colorectal liver metastases. Br J Surg.

[61] Boda-Heggemann J, Jahnke A, Chan MKH, Ghaderi Ardekani LS, Hunold P, Schäfer JP (2018). Direct dose correlation of MRI morphologic alterations of healthy liver tissue after robotic liver SBRT. Strahlenther Onkol.

[62] Yadav P, Kuczmarska-Haas A, Musunuru HB, Witt J, Blitzer G, Mahler P (2021). Evaluating dose constraints for radiation induced liver damage following magnetic resonance image guided stereotactic body radiotherapy. Phys Imaging Radiat Oncol.

[63] Ajdari A, Xie Y, Richter C, Niyazi M, Duda DG, Hong TS (2021). Toward personalized radiation therapy of liver metastasis: importance of serial blood biomarkers. JCO Clin Cancer Inform.

[64] Hall WA, van der Paulson ES, Heide UA, Fuller CD, Raaymakers BW, Lagendijk JJW (2019). The transformation of radiation oncology using real-time magnetic resonance guidance: a review. Eur J Cancer (Oxf, Engl: 1990).

[65] Boldrini L, Corradini S, Gani C, Henke L, Hosni A, Romano A (2021). MR-guided radiotherapy for liver malignancies. Front Oncol.

[66] Hehakaya C, van der Voort van Zyp JRN, Vanneste BGL, Grutters JPC, Grobbee DE, Verkooijen HM (2021). Early health economic analysis of 1.5 T MRI-guided radiotherapy for localized prostate cancer: decision analytic modelling. Radiother Oncol: J Eur Soc Ther Radiol Oncol.

[67] Parikh NR, Lee PP, Raman SS, Cao M, Lamb J, Tyran M (2020). Time-driven activity-based costing comparison of CT-guided versus MR-guided SBRT. JCO Oncol Pract.

[68] Leeman J. Stereotactic magnetic resonance guided radiation therapy (NCT04115254). https://clinicaltrials.gov/ct2/show/NCT04115254. Accessed 31 Aug 2021.

[69] Kirichenko A. Radiotherapy with iron oxide nanoparticles (SPION) on MR-Linac for primary & metastatic hepatic cancers (NCT04682847). https://clinicaltrials.gov/ct2/show/NCT04682847. Accessed 31 Aug 2021.

[70] Leclerc CGF. Adaptative MR-guided stereotactic body radiotherapy of liver tumors (RASTAF) (NCT04242342). https://clinicaltrials.gov/ct2/show/NCT04242342. Accessed 31 Aug 2021.

[71] University Health Network T. MR guidance for liver and pancreas (NCT03582189). https://clinicaltrials.gov/ct2/show/NCT03582189. Accessed 31 Aug 2021.

[72] University of Wisconsin M. OAR-based, dose escalated SBRT With real time adaptive MRI guidance for liver metastases (NCT04020276). https://clinicaltrials.gov/ct2/show/NCT04020276. Accessed 31 Aug 2021.

[73] Soni PD, Hartman HE, Dess RT, Abugharib A, Allen SG, Feng FY (2019). Comparison of population-based observational studies with randomized trials in oncology. J Clin Oncol: Off J Am Soc Clin Oncol.

