# Supplementary material for: MR-guided adaptive versus ITV-based stereotactic body radiotherapy for hepatic metastases (MAESTRO): a randomized controlled phase II trial
Source: Radiat Oncol. 2022 Mar 27;17:59. doi: 10.1186/s13014-022-02033-2 (PMC8958771; doi:10.1186/s13014-022-02033-2)
Supplement: Supplementary file 1 — Additional file 1. Study protocol. [file 13014_2022_2033_MOESM1_ESM.pdf]

CLINICAL TRIAL PROTOCOL

Magnetic Resonance-guided Adaptive Stereotactic Body Radiotherapy for Hepatic Metastases

- MAESTRO -

Phase-II-

Version 1.4, 19.11.2021

Protocolcode: RADONK-MAESTRO-2020

Reg.-Nr.: NCT05027711

Principal Investigator

PD Dr. Juliane Hörner-Rieber, Universitätsklinikum Heidelberg
Klinik für RadioOnkologie und Strahlentherapie (Czerny-Klinik)
Im Neuenheimer Feld 400
69120 Heidelberg
Germany

Confidential: This protocol contains confidential information and is intended solely for the guidance of the clinical investigation. This protocol may not be disclosed to parties not associated with the clinical investigation or used for any purpose without the prior written consent of the Principal Investigator.

Version 1.0	Erstellt	Überarbeitet V1.1	Überarbeitet V1.2
Am	15.10.20	17.12.20	14.09.21
Von	Hommertgen	Hommertgen	Hommertgen

1 TABLE OF CONTENTS

Abbreviations 5

Synopsis 10

Flowchart 14

1 Summary 15

2 Zusammenfassung 16

3 Introduction/Background 17

3.1 Scientific Background 17

3.2 Trial Rationale/ Justification 20

3.3 Benefit-Risk-Assessment 21

4 Trial Objectives 22

4.1 Primary Objective 22

4.2 Secondary Objectives 22

4.3 Explorative Objectives 23

5 Trial Description 23

5.1 Trial Design 23

5.2 Trial Duration and Schedule 23

6 Selection of Patients 24

6.1 Number of Patients 24

6.2 General Criteria for Patients‘ Selection 24

6.3 Inclusion Criteria 24

6.4 Exclusion Criteria 25

6.5 Criteria for Withdrawal 25

6.5.1 Withdrawal of Patients 25

6.5.2 Handling of Withdrawals 26

6.5.3 Premature Withdrawal of Patients from the Study 26

6.5.4 Replacement of Patients 26

6.5.5 Individual Termination Criteria during the Follow-up Phase 26

6.5.6 Premature End of Trial/ Withdrawal of the Whole Study 26

6.6 Prior and Concomitant Illnesses 26

6.7 Prior and Concomitant Treatments 27

7 MRI 27
8 Randomization 27
9 Radiation Therapy 28
9.1 Treatment Planning and Dose Prescription 28
9.2 Study Visits 31
10 Trial Methods 34
10.1 Assessment of Efficacy Parameters 35
10.1.1 Primary Endpoint: non-inferiority of MRgSBRT to ITV-SBRT regarding treatment-related toxicity 35
10.1.2 Secondary Endpoints 35
11 Plan for Treatment or Care after the Trial 39
12 Assessment of Safety 39
12.1 Adverse Events 39
12.2 Serious Adverse Events 40
13 Statistical Methods 41
13.1 Analysis Populations 41
13.2 Sample Size Calculation 41
13.3 Statistical Analysis 42
13.3.1 Primary Endpoint 42
13.3.2 Secondary Endpoints 42
13.3.3 Safety Analysis 43
14 Quality Assurance 43
14.1 SOP (Standard Operating Procedures) 43
14.2 Data Quality 43
15 Documentation 44
15.1 Data Management 44
15.2 Patient Identification List 44
15.3 Investigator Site File (ISF) 45
15.4 Data Storage 45
16 Reports, Publications 45
16.1 Final Report 45

16.2 Publications ... 45
17 Ethical, Legal and Administrative Aspects ... 46
17.1 Responsibilities of the Principal Investigator ... 46
17.2 Ethics Committee, DEGRO Expert Committee ... 47
17.3 Patient Information and Informed Consent ... 47
17.4 Patient Insurance ... 48
17.5 Data Protection and Medical Confidentiality ... 48
18 Funding ... 49
19 Amendments ... 49
20 Signatures ... 50
References: ... 52

Abbreviations

AE	Adverse Event
BED	Biologically Effective Dose
CBCT	Cone-Beam Computed Tomography
CT	Computed Tomography
CTCAE	Common Terminology Criteria for Adverse Events
CTV	Clinical Target Volume
(e)CRF	(electronic) Case Report Form
DIBH	Deep-inspiration Breath Hold
FAS	Full Analysis Set
FSI	First Subject In
GD	Gadolinium
GTV	Gross Tumor Volume
Gy	Gray
IGRT	Image-guided Radiotherapy
IMRT	Intensity-modulated Radiotherapy
ISF	Investigator Site File
ITT	Intention-to-treat
ITV	Internal Target Volume
ITV-SBRT	ITV-based Stereotactic Body Radiotherapy
LSI	Last Subject In
LSO	Last Subject Out
MR(I)	Magnetic Resonance (Imaging)
MRg(SB)RT	Magnetic Resonance-guided (Stereotactic Body) Radiotherapy
OAR	Organ at Risk
PP	Per-protocol
PTV	Planning Target Volume
RILD	Radiation-induced Liver Disease
RT	Radiotherapy
SAE	Serious Adverse Event
SBRT	Stereotactic Body Radiotherapy
SOP	Standard Operating Procedure
TMF	Trial Master File

Responsibilities

Principal Investigator (PI):

PD Dr. Juliane Hörner-Rieber
Department of Radiation Oncology
University of Heidelberg
Im Neuenheimer Feld 400
69120 Heidelberg, Germany
Phone: +49 6221-56-8202
Fax: +49 6221-56-5353
E-Mail: juliane.hoerner-rieber@med.uni-heidelberg.de

Co-Principal Investigator (PI):

Prof. Dr. Dr. Jürgen Debus
Department of Radiation Oncology
University of Heidelberg
Im Neuenheimer Feld 400
69120 Heidelberg, Germany
Phone: +49 6221-56-8202
Fax: +49 6221-56-5353
E-Mail: juergen.debus@med.uni-heidelberg.de

Principal Study coordinator:

Dr. Philipp Hoegen
Department of Radiation Oncology
University of Heidelberg
Im Neuenheimer Feld 400
69120 Heidelberg, Germany
Phone: +49 6221-56-8202
Fax: +49 6221-56-5353
E-Mail: philipp.hoegen@med.uni-heidelberg.de

Study coordinator:

Kevin Sun Zhang
Abteilung Radiologie (E010)
Deutsches Krebsforschungszentrum (DKFZ)
Im Neuenheimer Feld 280
69120 Heidelberg
Phone: +49 6221 42-2611
Fax: +49 6221 42-2513
E-Mail: kevin.zhang@dkfz-heidelberg.de

Study coordinator:

PD Dr. Patrick Naumann
Klinik für Radioonkologie und Strahlentherapie
Universität Heidelberg
Im Neuenheimer Feld 400, 69120 Heidelberg
Tel.: +49-6221-56 8202
Fax: +49-6221-56 5353
E-Mail: patrick.naumann@med.uni-heidelberg.de

Study coordinator:

Dr. Fabian Weykamp
Klinik für Radioonkologie und Strahlentherapie
Universität Heidelberg
Im Neuenheimer Feld 400, 69120 Heidelberg
Tel.: +49-6221-56 8202
Fax: +49-6221-56 5353
E-Mail: fabian.weykamp@med.uni-heidelberg.de

Study coordinator: **Dr. Sebastian Regnery**
Klinik für Radioonkologie und Strahlentherapie
Universität Heidelberg
Im Neuenheimer Feld 400, 69120 Heidelberg
Tel.: +49-6221-56 8202
Fax: +49-6221-56 5353
E-Mail: sebastian.regnery@med.uni-heidelberg.de

Study coordinator: **PD Dr. Stefan Körber**
Klinik für Radioonkologie und Strahlentherapie
Universität Heidelberg
Im Neuenheimer Feld 400, 69120 Heidelberg
Tel.: +49-6221-56 8202
Fax: +49-6221-56 5353
E-Mail: stefan.koerber@med.uni-heidelberg.de

Study coordinator: **Dr. Oliver Sedlaczek**
Abteilung für Radiologie (E010)
Deutsches Krebsforschungszentrum
Im Neuenheimer Feld 280
69120 Heidelberg
Phone: +49 6221 42-2611
Fax: +49 6221 42-2513
E-Mail: o.sedlaczek@dkfz.de

Study coordinator: **Prof. Dr. Stefan Delorme**
Abteilung für Radiologie (E010)
Deutsches Krebsforschungszentrum
Im Neuenheimer Feld 280
69120 Heidelberg
Phone: +49 6221 42-2611
Fax: +49 6221 42-2513
E-Mail: s.delorme@dkfz.de

Study coordinator: **Prof. Dr. med. Heinz-Peter Schlemmer**
Abteilung für Radiologie (E010)
Deutsches Krebsforschungszentrum
Im Neuenheimer Feld 280
69120 Heidelberg
Phone: +49 6221 42-2611
Fax: +49 6221 42-2513
E-Mail: h.schlemmer@dkfz.de

Study coordinator: **Prof. Dr. med. Dipl.-Phys. Karim-Maximilian Niyazi**
Klinik und Poliklinik für Strahlentherapie und Radioonkologie
Marchioninistr. 15
81377 München
Phone: +49 89-4400 73770
Fax: +49 89-4400 76770
E-Mail: maximilian.niyazi@med.uni-muenchen.de

Study coordinator: **PD Dr. med. Dr. med. univ. Stefanie Corradini**
Klinik und Poliklinik für Strahlentherapie und Radioonkologie
Marchioninistr. 15
81377 München
Phone: +49 89-4400 73770
Fax: +49 89-4400 76770
E-Mail: stefanie.corradini@med.uni-muenchen.de

Study medical physicist:

Dr. Sebastian Klüter
Klinik für Radioonkologie und Strahlentherapie
Universität Heidelberg
Im Neuenheimer Feld 400, 69120 Heidelberg
Tel.: +49-6221-56 8202
Fax: +49-6221-56 5353
E-Mail: sebastian.klueter@med.uni-heidelberg.de

Study medical physicist:

Carolin Buchele
Klinik für Radioonkologie und Strahlentherapie
Universität Heidelberg
Im Neuenheimer Feld 400, 69120 Heidelberg
Tel.: +49-6221-56 8202
Fax: +49-6221-56 5353
E-Mail: carolin.buchele@med.uni-heidelberg.de

Study medical physicist:

Dr. Katharina Maria Paul
Klinik für Radioonkologie und Strahlentherapie
Universität Heidelberg
Im Neuenheimer Feld 400, 69120 Heidelberg
Tel.: +49-6221-56 8202
Fax: +49-6221-56 5353
E-Mail: KatharinaMaria.Paul@med.uni-heidelberg.de

Head of Clinical Trial Office RadioOncology:

Dr. Adriane Hommertgen
Department of Radiation Oncology
University of Heidelberg
Im Neuenheimer Feld 400
69120 Heidelberg, Germany
Phone: +49 6221-56-34091
Fax: +49 6221-56-8968
E-Mail: adriane.hommertgen@med.uni-heidelberg.de

Study assistance and documentation:

Studienambulanz Radioonkologie
Department of Radiation Oncology
University of Heidelberg
Im Neuenheimer Feld 400
69120 Heidelberg, Germany
Phone: +49 6221-56-38613/-37748/-36318/-32682
Fax: +49 6221-56-1979
E-Mail: studien.radonk@med.uni-heidelberg.de

Trial biometrician:

Dr. Johannes Krisam
Institute of Medical Biometry and Informatics,
University of Heidelberg
Im Neuenheimer Feld 130.3
69120 Heidelberg, Germany
Phone: +49 6221-56-34516
E-mail: krisam@imbi.uni-heidelberg.de

Trial representative biometrician:

Christopher Büsch
Institute of Medical Biometry and Informatics,
University of Heidelberg
Im Neuenheimer Feld 130.3
69120 Heidelberg, Germany
Phone: +49 6221-56-5945
E-mail: buesch@imbi.uni-heidelberg.de

IT:

Matthias Dostal
Department of Radiation Oncology
University of Heidelberg
Im Neuenheimer Feld 400
69120 Heidelberg, Germany
Tel.: +49 6221-56-37656
Fax: +49 6221-56-8968
E-Mail: matthias.dostal@med.uni-heidelberg.de

Synopsis

**Titel**	Magnetic Resonance-guided Adaptive Stereotactic Body Radiotherapy for Hepatic Metastases
**Acronym**	MAESTRO
**Trial design**	Multi-center, three-armed prospective phase II study
**Principal Investigator**	PD Dr. Juliane Hörner-Rieber
**Study Coordinator**	Dr. Philipp Hoegen
**Clinical Trial Office**	Dr. Adriane Hommertgen/ Dr. Cornelia Jäkel
**Study nurses**	Studynurse Team *Studienambulanz Radioonkologie*
**Number of Patients**	To be assessed for eligibility: (n =90)<br>To be assigned to the trial, i.e. recruited: (n =82)<br>To be analyzed: (n =82)
**Inclusion criteria**	• confirmed underlying solid malignant tumor (no germ cell tumor, leukemia, lymphoma)<br>• 1-3 hepatic metastases confirmed by pre-therapeutic MRI<br>• indication for SBRT of 1-3 hepatic metastases<br>• maximum diameter of each hepatic metastasis ≤ 5 cm (in case of 3 metastases: sum of diameters ≤ 12 cm)<br>• age ≥ 18 years of age<br>• Karnofsky Performance Score ≥ 60%<br>• ability to lie still on a linac table for at least one hour<br>• ability to hold one's breath for more than 25 seconds<br>• for women with childbearing potential, adequate contraception<br>• ability of subject to understand character and individual consequences of the clinical trial<br>• written informed consent (must be available before enrolment in the trial)
**Exclusion criteria**	• refusal of the patients to take part in the study<br>• patients with liver cancer (e.g. HCC, CCC)<br>• patients after liver transplantation<br>• impairment of liver function to an extent contraindicating radiotherapy (to the discretion of the treating radiation oncologist)<br>• active and acute hepatic/biliary infection (e.g. hepatitis, cholangitis, cholecystitis)<br>• previous radiotherapy of the upper abdomen with dose to the gastrointestinal tract preventing safe liver SBRT<br>• patients who have not yet recovered from acute toxicities of prior therapies<br>• claustrophobia<br>• pregnant or lactating women<br>• contraindications against performing contrast-enhanced MRI scans (pacemakers, other implants making MRI impossible, allergy to gadolinium (GD)-based contrast agent)

	• participation in another competing clinical study or observation period of competing trials
**Therapy**	Arms A and C: Magnetic Resonance-guided Stereotactic Body Radiotherapy (MRgSBRT)<br>Arm: B: ITV (Internal target volume)-based Stereotactic Body Radiotherapy (ITV-SBRT)<br>If a biologically effective dose (BED) of ≥ 100 Gy is achievable using an ITV, patients will be randomized to either MRgSBRT (Arm A) or ITV-SBRT (Arm B).<br>If a BED of ≥ 100 Gy cannot be achieved using an ITV concept (e.g. due to OAR constraints), patients will be treated in arm C using MRgSBRT with the highest achievable dose as deemed appropriate by the treating radiation oncologist.
**Mode of Radiotherapy**	photons
**Objectives of Clinical Trial**	<u>Primary Endpoint</u><br>Occurrence of gastrointestinal and hepatobiliary toxicity CTCAE III° or higher (non-inferiority assessment of MRgSBRT vs. ITV-SBRT) assessed within the first year with NCI CTCAE Version 5.0<br><u>Secondary Endpoints:</u><br>• Comparison of all three treatment groups with respect to the primary endpoint<br>• Rate of patients/metastases in which a BED ≥ 100 Gy can be achieved<br>• BED increase of ≥ 5 Gy for patients treated with MRgSBRT compared to ITV-SBRT<br>• Local control (treated lesion), locoregional control (liver), distant tumor control<br>• Progression-free survival and overall survival<br>• Toxicity according to CTCAE V5.0, treatment-related toxicity, quality of life<br>• Morphological and functional changes in MRI after radiation and as a comparison between the two different SBRT techniques<br>• Acquisition and comparison of different MRI sequences<br>• Collection of treatment plan and irradiation parameters as well as imaging data and quality assurance results for further analysis and planning of follow-up projects.<br><u>Explorative Objectives:</u><br>Longitudinal evaluation of clinical patient-related, tumor-related parameters on radiological imaging, and blood tests including assessment of potential biomarkers. Analysis of available tumor samples for genetic mutations.<br>**Assessment of safety:**<br>For the safety analysis all SAEs will be analyzed via descriptive statistical methods in the safety population. The safety analysis includes calculation of frequencies and rates of complications and serious adverse events together with corresponding 95% confidence intervals.
**Sample size calculation**	The sample size calculation is based on the primary comparison of the rates of gastrointestinal and hepatobiliary toxicity CTCAE of grade III or higher between the two treatment groups (arm A and B). A total of 62 patients are needed to assess non-inferiority by means of a Farrington-Manning test of MRgSBRT to ITV-

	SBRT with a power of 80% at a one-sided significance level of 10%, with allowance for 5% loss to follow-up and with the use of a clinically relevant non-inferiority margin of δ =10%, when assuming toxicity rates of $p_{MRgSBRT}$=2% and $p_{ITV\text{-}SBRT}$=5%. It is expected that using a shifted Mantel-Haenszel type test for non-inferiority adjusting for the factor centre will yield an increased power.<br><br>To achieve a comparable arm C (BED < 100 Gy with ITV-SBRT) for the secondary objectives, the study will recruit patients until at least 20 analyzable patients are present in arm C. Due to the fact that patients in arm C are still treated with the best possible treatment, this approach is ethically justifiable.<br><br>Sample size calculation was performed using PASS 16.0.3.
**Statistical analysis**	The primary analysis will be based on the full analysis set (FAS) including all enrolled patients according to the intention to treat principle. The hypotheses for the primary analysis are<br>$H_0: p_{\text{MRgSBRT}} - p_{\text{ITV-SBRT}} \geq \delta \;\; vs. \; H_1: p_{\text{MRgSBRT}} - p_{\text{IITV-SBRT}} < \delta$<br>($\delta$ =10% non-inferiority margin), where p_{MRgSBRT} and $p_{\text{ITV-SBRT}}$ are the probabilities for an occurrence of a gastrointestinal and hepatobiliary toxicity CTCAE of grade III or higher for the MRgSBRT (arm A) and the ITV-SBRT (arm B) group, respectively. Non-inferiority of MRgSBRT as compared to ITV-SBRT will be tested at a one-sided significance level of α=0.1 using a Mantel-Haenszel type test for non-inferiority adjusting for the stratum "centre". Missing values for the primary outcome will be imputed using multiple imputation. Sensitivity analyses will be performed by means of conducting an analysis for the per-protocol (PP) population (based on those patients without major protocol violation).<br><br>Analysis of the secondary endpoints will also be based on the FAS. In order to compare all three treatment groups with regard to the primary endpoint, a (descriptive) Chi-squared test will be used. The secondary endpoints distant tumor control, overall survival and progression-free survival will be analyzed using Kaplan-Meier-Curves. The 1-year and 2-year survival rates as well as the median survival rate will be provided alongside two-sided 95%-confidence intervals. Descriptive pairwise logrank tests stratified for "centre" will be conducted to compare all three treatment groups. The secondary endpoints local and locoregional control will be analyzed via cumulative incidence functions, taking the competing event death into account.<br>The other secondary endpoints and the patients' characteristics will be displayed by descriptive measures. Descriptive pairwise comparisons between all three treatment groups will be performed. Continuous variables will be described using number non-missing values, mean, standard deviation, median, Q1, Q3, minimum and maximum. For binary or categorical variables absolute and relative frequencies will be provided. Furthermore, two-sided 95%-confidence intervals will be calculated.<br><br>The safety analysis is based on the safety set including all patients who received one of the study treatments, and includes calculation of frequencies and rates of adverse and serious adverse events together with corresponding 95%-confidence intervals.<br><br>Further details of the analysis will be specified in the statistical analysis plan (SAP) which will be finalized before database closure. All analyses will be done using SAS version 9.4 or higher.
**Trial duration**	Total trial duration: 60 months<br>Recruitment phase: 36 months<br>Minimal Follow-up: 24 months

	FSI (First subject in):<br>LSI (Last subject in):<br>LSO (last subject out):	01.02.2021<br>31.01.2024<br>31.01.2026
**Trial centers**	University Hospital Heidelberg, University Hospital Munich (LMU)	

Flowchart

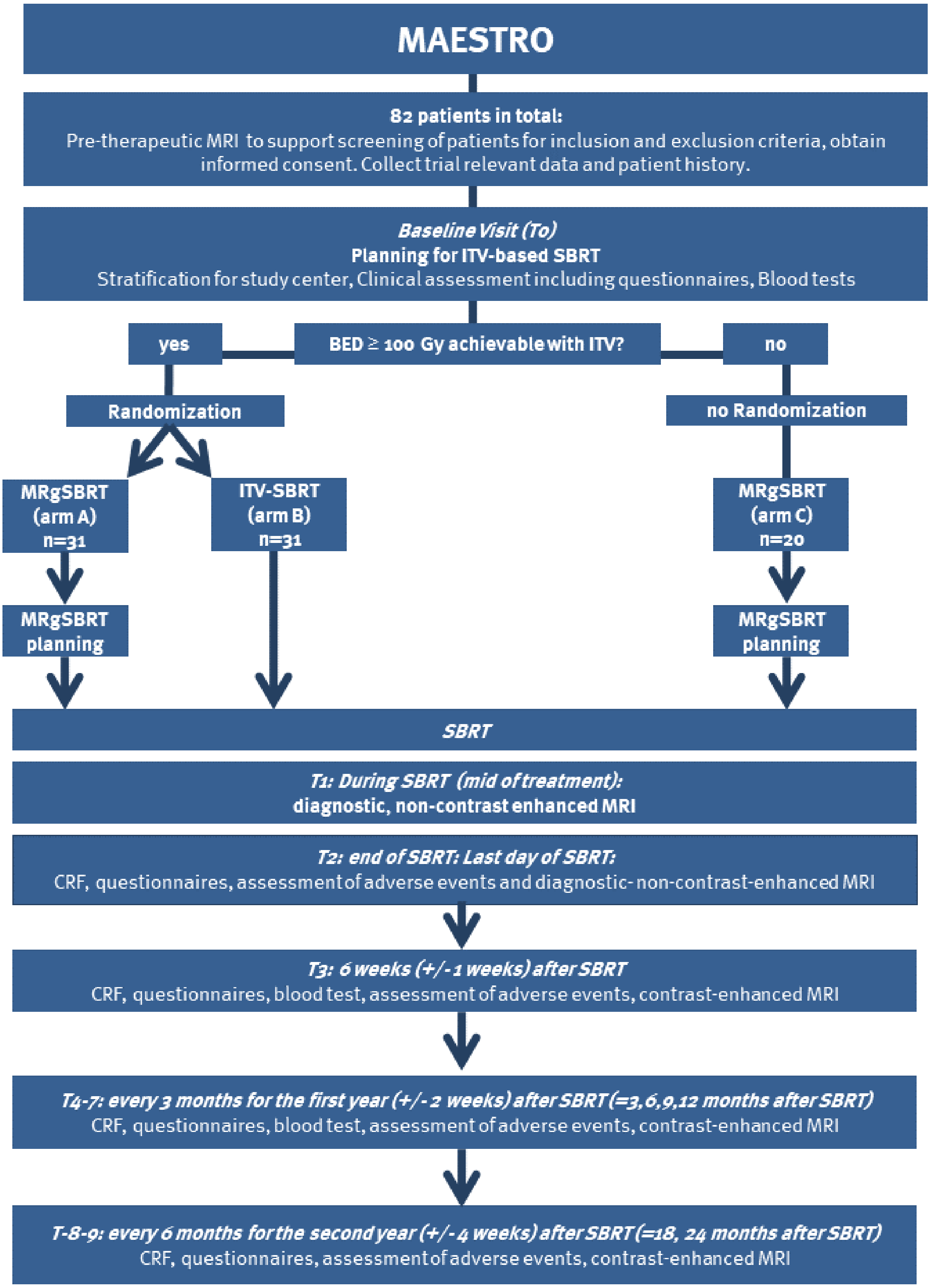
MAESTRO
82 patients in total:
Pre-therapeutic MRI to support screening of patients for inclusion and exclusion criteria, obtain informed consent. Collect trial relevant data and patient history.
Baseline Visit (T0)
Planning for ITV-based SBRT
Stratification for study center, Clinical assessment including questionnaires, Blood tests
yes
BED ≥ 100 Gy achievable with ITV?
no
Randomization
no Randomization
MRgSBRT (arm A) n=31
ITV-SBRT (arm B) n=31
MRgSBRT (arm C) n=20
MRgSBRT planning
MRgSBRT planning
SBRT
T1: During SBRT (mid of treatment):
diagnostic, non-contrast enhanced MRI
T2: end of SBRT: Last day of SBRT:
CRF, questionnaires, assessment of adverse events and diagnostic-non-contrast-enhanced MRI
T3: 6 weeks (+/-1 weeks) after SBRT
CRF, questionnaires, blood test, assessment of adverse events, contrast-enhanced MRI
T4-7: every 3 months for the first year (+/-2 weeks) after SBRT (=3,6,9,12 months after SBRT)
CRF, questionnaires, blood test, assessment of adverse events, contrast-enhanced MRI
T-8-9: every 6 months for the second year (+/-4 weeks) after SBRT (=18, 24 months after SBRT)
CRF, questionnaires, assessment of adverse events, contrast-enhanced MRI

1 Summary

Stereotactic body radiotherapy (SBRT) is an established local treatment method for patients with hepatic oligometastases. Liver metastases often occur in close proximity to radiosensitive organs at risk (OARs). This limits the possibility to apply sufficiently high doses needed for optimal local control. MR-guided radiotherapy (MRgRT) is expected to hold potential to improve hepatic SBRT by offering superior soft-tissue contrast for enhanced target identification as well as the benefit of daily real-time adaptive treatment. The MAESTRO trial therefore aims to assess the potential advantages of adaptive, gated MR-guided SBRT (MRgSBRT) compared to conventional SBRT at a standard linac using an ITV (internal target volume) approach (ITV-SBRT).

This trial will be conducted as a prospective, randomized, three-armed phase II study in 82 patients with hepatic metastases (solid malignant tumor, 1-3 hepatic metastases confirmed by magnetic resonance imaging (MRI), maximum diameter of each metastasis ≤ 5 cm (in case of 3 metastases: sum of diameters ≤ 12cm), age ≥ 18 years, Karnofsky Performance Score ≥ 60%). If a biologically effective dose (BED) ≥ 100 Gy is feasible based on ITV-based planning, patients will be randomized to either MRgSBRT (Arm A) or ITV-based SBRT (Arm B). If a lesion cannot be treated with a BED ≥ 100 Gy, the patient will be treated in Arm C with MRgSBRT at the highest possible dose.
Primary endpoint is the non-inferiority of MRgSBRT at the MRIdian Linac® system compared to ITV-based SBRT regarding hepatobiliary and gastrointestinal toxicity CTCAE III° or higher. Toxicity is not expected to be increased for hepatic MRgSBRT compared to ITV-SBRT.

Secondary outcomes investigated are local, locoregional and distant tumor control, progression-free survival, overall survival, possible increase of BED using MRgSBRT if BED is limited with conventional ITV-based SBRT, treatment-related toxicity, quality of life, dosimetric parameters of radiotherapy plans as well as morphological and functional changes in MRI. Potential prognostic biomarkers will also be evaluated.

MRgSBRT is known to be both highly cost- and labor-intensive. The MAESTRO trial therefore aims to provide initial evidence for the dosimetric and possible consecutive clinical benefit of MR-guided, on-table adaptive and gated SBRT for dose escalation in critically located hepatic metastases adjacent to radiosensitive OARs.

2 Zusammenfassung

Die stereotaktische Bestrahlung (SBRT) stellt eine etablierte Therapiemethode bei hepatischer Oligometastasierung dar. Lebermetastasen werden oftmals in direkter Nähe zu radiosensitiven Risikoorganen (bspw. Duodenum, Colon) diagnostiziert. Daher kann in diesen Fällen die für eine optimale lokale Kontrolle erforderliche Dosis nicht appliziert werden. Die MR-geführte Radiotherapie (MRgRT) bietet das Potential durch bessere Zielvolumenabgrenzung aufgrund des überlegenen Weichgewebekontrasts sowie durch die Möglichkeit der täglichen Adaptation in Echtzeit deutliche Verbesserungen der Leber-SBRT zu ermöglichen.

In der MAESTRO Studie soll daher die Nicht-Unterlegenheit der adaptiven MR-geführten SBRT (MRgSBRT) in Bezug auf die Toxizität im Vergleich zur SBRT an einem konventionellen Linearbeschleuniger mit Internal-Target-Volume (ITV)-Konzept geprüft werden (ITV-SBRT).

In dieser prospektiven, randomisierten, dreiarmigen Phase-II-Studie werden 82 Patienten mit hepatischen Metastasen eines soliden Tumors (1-3 durch Magnetresonanztomographie (MRT) bestätigte Metastasen, maximaler Metastasendurchmesser 5 cm, bei 3 Metastasen kumulativ maximal 12 cm Durchmesser, Alter ≥ 18 Jahre, Karnofsky Index ≥ 60%) mittels SBRT behandelt. Ist mittels ITV eine biologisch effektive Dosis (BED) von 100 Gy erreichbar, so werden Patienten auf MRgSBRT (Arm A) und ITV-Konzept (Arm B) randomisiert. Ist eine BED von 100 Gy nicht möglich, erfolgt die Behandlung mit der höchstmöglichen Dosis mittels MRgSBRT (Arm C).

Primärer Endpunkt ist die Nichtunterlegenheit der MR-geführten SBRT bzgl. hepatobiliärer und gastrointestinaler Toxizität ab CTCAE III° im Vergleich zur ITV-basierten SBRT an einem konventionellen Linearbeschleuniger. Im Vergleich zur ITV-basierten SBRT wird keine höhere Toxizität für die MRgSBRT erwartet.

Als sekundäre Endpunkte werden die Lokalkontrolle, das progressionsfreie Überleben, das Gesamtüberleben, die hepatobiliäre und gastrointestinale Toxizität, eine mögliche Dosiseskalation mittels MRgSBRT in Arm C, die Lebensqualität, dosimetrische Parameter der Bestrahlungspläne sowie bildgebende Veränderungen erhoben. In einem translationalen Ansatz werden zudem potentielle Biomarker evaluiert.

Die MRgSBRT ist sowohl kosten- als auch personalintensiv. Ziel der MAESTRO Studie ist es daher, den klinischen Vorteil der MR-geführten SBRT mit „on-table“-Adaptation und Gating zur Ermöglichung einer Dosiseskalation bei kritisch lokalisierten Lebermetastasen nahe strahlensensibler Risikoorgane aufzuzeigen.

3 Introduction/Background

3.1 Scientific Background

Standard therapy for patients with hepatic oligometastases is surgical resection. Hepatic metastasectomy has been performed for more than three decades with 5-year survival rates of 50-60% and up to 20% long-term survivors [1-4]. However, only 15-20% of patients with hepatic oligometastases are initially eligible for such a radical surgical approach due to an unresectable tumor location, inadequate hepatic reserve or other comorbidities [5, 6]. For the majority of patients who are not amenable for surgery, alternative liver-directed therapies are offered for providing local control, e.g. radiofrequency ablation, transarterial chemoembolization, cryoablation or stereotactic body radiotherapy (SBRT).

Radiation therapy as low-dose whole-liver irradiation is well established in the palliative treatment of patients with symptoms from diffuse liver metastases with the aim of achieving pain relief and improving quality of life [7, 8]. However, for truly eradicating hepatic metastases higher doses are needed which cannot be delivered to the whole liver due to its relative radiosensitivity. Radiation-induced liver disease (RILD) is a feared complication following hepatic irradiation [9, 10]. Its pathogenesis includes venoocclusive hepatic fibrosis [11]. Fibrosis is mediated by growth factors and other cytokines such as Tumor Necrosis Factor alpha (TNF-α) and Transforming Growth Factor beta (TGF-β) [12]. Release of pro-inflammatory interleukines (IL) (e.g. IL-6, IL-13, IL-17, IL-33) and anti-inflammatory interleukines (e.g. IL-10) play a major role in this inflammatory process [13]. Nevertheless, due to its parallel architecture model of radiobiology, the liver can tolerate high doses to small volumes as long as the mean dose to the uninvolved hepatic tissue is kept below the threshold above which severe RILD is observed [14].

More recently, technological advances in target definition, treatment planning and methods of image guidance have enabled precise local ablative treatment of small hepatic lesions by applying SBRT. SBRT allows for safe delivery of large single doses of highly conformal radiation with steep dose gradients to the surrounding healthy tissue over a limited number of fractions. Due to its spatial precision, SBRT permits the administration of tumoricidial radiation doses to hepatic metastases, while sparing organs at risk (OARs) including the surrounding healthy hepatic tissue and hence toxicity. Several retrospective and prospective series reported 1- and 3-year local control (LC) rates of 56-100% and 45-100% following SBRT for liver metastases, respectively [15-20]. Similar to pulmonary SBRT, a dose-response relationship is assumed for hepatic SBRT [15, 20]. A recent meta-analysis by Ohri et al. reported significantly superior 3-year LC of 93% for hepatic metastases treated with biologically effective doses (BEDs) exceeding 100 Gy than for those irradiated with BEDs < 100 Gy with 3-year LC of only 65% [20]. However, metastases in close

proximity to organs at risk (small bowel, duodenum) as well as large central metastases often cannot be treated with sufficiently high doses due to the increased risk of toxicity [21-23]. The applied maximum dose to the stomach and the intestine is known to significantly correlate with the risk of severe gastroduodenal toxicity [24]. Hence, currently lower total doses have to be applied for such lesions reducing the possibility of long-time local control.

Abdominal organs like the liver are subject to movement caused by breathing and positional drifts in the body of several centimeters during treatment [25-27]. Motion management strategies are therefore crucial for safe application of high-dose liver SBRT. Clinically established strategies for motion compensation include breath hold techniques or continuous irradiation in free breathing with an internal target volume (ITV) [17]. The ITV concept, which is most widely used, accounts for tumor movement by incorporating tumor motion on several breathing phases assessed by a 4-dimensional (4D) CT [28].

In routine clinical practice, low dose computed tomography scans ("cone-beam CT" (CBCT)) are generally used for daily imaging of patient positioning, tumor location and alterations in patient anatomy (image-guided radiotherapy, IGRT). However, image-guidance for hepatic SBRT is challenged by the lack of tumor visibility in CBCT images due to their limited soft tissue contrast. Consequently, for precisely targeting hepatic metastases, some centers have patients undergo invasive implantation of fiducial markers in the liver near the tumor for topographic orientation [29]. Furthermore, daily application of CBCTs is accompanied by the exposure of an additional amount of dose, which in turn might even lead to an increased risk of secondary malignancies [30].

For treatment planning, magnetic resonance imaging (MRI) has gained a fundamental role in daily clinical practice, especially with regard to detecting and characterizing abdominal malignancies, such as hepatobiliary cancer [31]. MRI with its superior soft tissue contrast enhances tumor delineation by enabling superior distinction between cancerous and normal tissues [32]. Additionally, functional MRI with modern, optimized sequences allows for non-invasive assessment of tissue perfusion, diffusion or cellular density, exceeding the morphological characterization of conventional MRI [33, 34].

For example, diffusion-weighted imaging (DWI) not only facilitates the identification of diffusion-altered tumor tissue like hepatic metastases from surrounding healthy tissue, but also enables quantitative evaluation of suspicious lesions e.g. by using the apparent-diffusion coefficient (ADC) (Figure 1). The ADC has been shown to be predictive for treatment response to radiotherapy in hepatic and rectal lesions [35, 36] and further correlates with cellularity [37, 38].

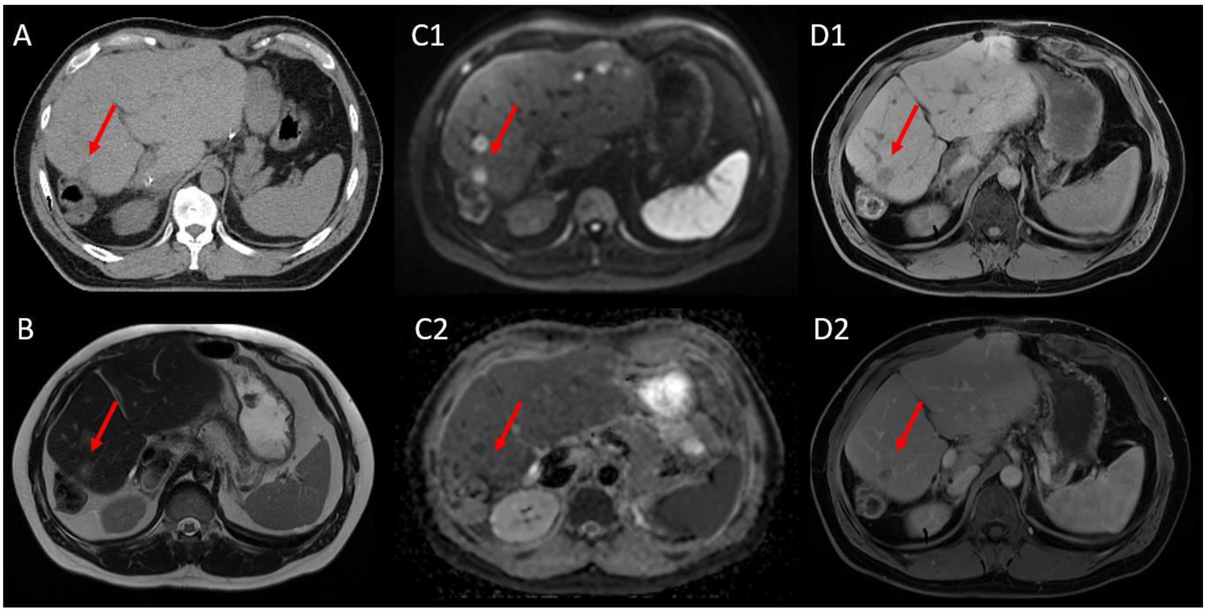

Figure 1 Treatment planning CT (A) and MRI (B-D): A patient with multiple liver metastases. A: native CT scan. B: T2 weighted sequence. C1: Diffusion-weighted Imaging (DWI) at the setting of b=900mm²/s. C2: Apparent Diffusion Coefficient (ADC at b50,900mm²/s). D: T1 weighted sequences with D1: native phase and D2: portal venous phase. Red arrows = demarcation of the target lesion.

MR-guided radiotherapy has recently become clinically available, providing an excellent soft tissue contrast for precise detection of tumor position and potential daily changes in patient anatomy without additional radiation dose [39, 40]. MR-guidance further offers the possibility to visualize tumor volume and nearby OARs during the whole treatment session (cine MRI). Safety margins and hence the irradiated volume can be decreased for hepatic MR-guided SBRT thereby reducing the risk of potential toxicity [41, 42]. Hepatic SBRT of smaller target volumes might offer the possibility of dose escalation for increasing local control.

MR-guided radiotherapy further allows for online plan adaptation in response to specific changes in tumor and OAR anatomy that may occur during the course of treatment. Conventionally, one treatment plan is generated based on patient anatomy during planning CT imaging. However, significant organ motion is known to occur in-between different treatment fractions e.g. due to varying filling of hollow organs or tumor shrinkage in response to therapy (interfractional organ motion) [43, 44]. MR-guided radiotherapy now enables daily imaging of sufficient quality to permit immediate plan adjustments in response to the anatomy of the day, while the patient keeps lying on the treatment couch [39, 45-47]. Online plan adaptation allows for superior protection of OARs and offers the possibility for dose escalation hereby potentially improving local control rates [42, 46]. Particularly for hepatic metastases located near the liver margin, superior visualization before and during irradiation allows for confidence to treat with high doses near organs such as bowel or stomach, where position uncertainty could lead to a dose that exceeds OAR tolerance [48]. With real-time imaging, treatment plans for hepatic metastases can be adapted if needed and RT doses can potentially be better personalized.

Consequently, by hybrid MRI-Linear accelerators, oncologic treatment with radiotherapy might improve treatment outcome both with regard to tumor response and treatment related side effects, as for the possibility of monitoring treatment related changes by different morphologic and functional MRI sequences.

Up to now, studies for MR-guided SBRT (MRgSBRT) for hepatic lesions are scarce with only two retrospective analyses including 26 and 29 patients as well as one case report [41, 48, 49]. However, MRgSBRT is very staff-intense and time-consuming compared to standard CT-guided hepatic SBRT [50, 51]. Hence, prospective studies are needed to assess which patients profit most from this new technique. The aim of the present study is therefore to evaluate potential benefits of MRgSBRT compared to ITV-based SBRT as one of the current state-of-the-art standard techniques. Non-inferiority of MRgSBRT compared to standard ITV-based SBRT for hepatic metastases will be evaluated in respect to gastrointestinal and hepatobiliary toxicity ≥ grade III. Special attention will be paid to whether MRgSBRT offers potential for dose escalation in case of critical proximity of hepatic metastases to gastrointestinal OARs, currently limiting application of a sufficient BED of ≥ 100 Gy in certain cases.

Few studies have examined potential predictive biomarkers for treatment response after hepatic SBRT. Hong et. al examined the potential effect of genetic aberrations (BRAF, EGFR, HER2, KRAS, NRAS, PIK3CA, TP53) and found tumors with KRAS and TP53 mutations to be particularly radioresistant, leading to decreased 1-year local control (20,0 % vs. 69,2 %, p = 0,001) [52]. The same group identified the plasma concentrations of IL-6, IL-8 and TNF-α as further potential predictors of local failure. Initial and mid-treatment plasma levels of IL-6 were predictive for reduced local control (p=0,01) [53]. This study was mainly limited by low radiation doses (BED 42-100 Gy), not sufficient for optimal local control according to present guidelines. Therefore, these data is not applicable to SBRT of hepatic metastases with higher, ablative doses. Predictability of individual side effects and tumor response in ablative hepatic SBRT remains an unmet need.

3.2 Trial Rationale/ Justification

Hepatic SBRT is a well-established local treatment method for technically or medically inoperable hepatic metastases [17, 54]. However, clinicians are often restricted in the utilization of hepatic SBRT due to dose limitations of the uninvolved liver and nearby OARs (e.g. small bowel, stomach, kidney). MR-guided radiotherapy with its superior soft-tissue contrast is believed to facilitate the precise detection of tumor position and interfractional changes in patient anatomy [39, 55]. Respiratory gating at the MR-Linac enables real-time visualization of the tumor as well as synchronization of beam delivery to the patient's breathing [56]. Therefore, safety margins and thus the irradiated volume can possibly be decreased with MRgSBRT in comparison to ITV-based

SBRT, reducing the risk of treatment-associated toxicity. Hepatic MRgSBRT of smaller target volumes might further offer the possibility of dose escalation for increasing local control. To our knowledge, only two retrospective analyses including 26 and 29 patients as well as one case report about hepatic MR-guided SBRT using a MR-Linac can be found in literature [41, 48, 49]. As MR-guided adaptive SBRT is very staff intense and time consuming compared to standard ITV-based SBRT, prospective studies are needed to demonstrate the expected benefits of MR-guided adaptive SBRT.

According to current guidelines, dose and fractionation depend solely on location, size and proximity to OARs [17]. Biomarkers predicting response or potential toxicity have not been established in clinical routine. Identification of sub-cohorts with particularly radioresistant tumors or particularly radiosensitive normal tissue is crucial to enable for future personalized radiotherapy.

3.3 Benefit-Risk-Assessment

At Heidelberg University Hospital, patients diagnosed with hepatic oligometastases or oligoprogression are treated within the National Center of Tumor Diseases (NCT), a multidisciplinary cooperation, where patients with liver metastases from different primary tumors are thoroughly discussed and treatment approaches are decided in tumor conferences consisting of gastroenterologists, abdominal surgeons, oncologist, radiologists, radiation oncologists as well as pathologists. This concept assures that all patients receive the optimal treatment and are treated to the limit of professional and scientific possibilities.

In this context, the Department of Radiation Oncology performs intra- and extracranial stereotactic radiotherapy in several hundred patients per year. The department has high experience and expertise in radiation treatments, and extracranial stereotactic body radiotherapy (SBRT) is established as a standard therapy approach for different types of metastases and primary tumors since 1997 [57-59]. Indeed, the Department of Radiation Oncology in Heidelberg was one of the first worldwide to perform stereotactic body radiotherapy of liver lesions. Since then, several hundred patients have been treated successfully with hepatic SBRT. Excellent treatment results have been published in several international peer-reviewed journals [58, 60-64]. During the last decade, SBRT has become a standard local treatment for hepatic oligometastases or oligoprogression [17, 54]. For example, the current versions of both NCCN (National Comprehensive Cancer Network) guidelines for colon and rectal cancer recommend SBRT as local ablative therapy for syn- or metachronous irresectable hepatic oligometastases [65, 66].

As intensely described above, MR-guided hepatic SBRT, which allows for superior visualization of intra- and interfractional anatomy changes, enables more precise and tailored local ablative radiotherapy compared to conventional techniques. Daily online plan adaptation allows for superior sparing of surrounding healthy tissue, while dose escalation in close proximity to critical OARs becomes feasible. The Department of Radiation Oncology of Heidelberg University was chosen out of several applicants by the German Research Foundation to clinically and scientifically evaluate the potential of MR-guided radiotherapy. The German Research Foundation therefore financed one of the first MR-Linac machines available worldwide (MRIdian® Linac from ViewRay) in the Department of Radiation Oncology at University Hospital Heidelberg. Potential negative aspects of MR-guided hepatic SBRT might include reduced patient comfort during treatment caused by limited space within the MR-Linac bore as well as well as increased noise pollution of the MRI during radiotherapy. From an institutional point of view, MR-guidance requires increased personnel expenditures and is time-consuming compared to conventional techniques. However, by participating in this trial, patients with hepatic oligometastases or oligoprogression will have the chance to be treated with advanced and modern, state-of-the-art hepatic SBRT techniques with a possibly increased probability of sufficiently high dose in the treated metastases and are provided intensive follow-up visits including regularly performed high-quality MRI.

4 Trial Objectives

4.1 Primary Objective

The primary endpoint is defined as the occurrence of treatment-related gastrointestinal or hepatobiliary CTCAE V5.0 toxicity of grade III or higher assessed within the first year. The primary aim of the study is the assessment of non-inferiority of MRgSBRT to ITV-SBRT regarding the primary endpoint, assuming a non-inferiority margin of $\delta = 10\%$. Toxicity of CTCAE III° or higher is generally low, between <1% [60] and 7.7% [48] according to published literature. Therefore, a non-inferiority-margin of δ=10% is deemed adequate.

4.2 Secondary Objectives

Secondary endpoints are:

- toxicity according to CTCAE V5.0 in all three treatment groups
- quality of life according to EORTC QLQ C-30 and EORTC QLQ LMC-21
- local control (LC)
- locoregional control

- distant tumor control
- progression-free survival (PFS)
- overall survival (OS)
- comparison of efficacy of ITV-based SBRT compared to MRgSBRT with respect to toxicity and local control
- morphological and functional changes in MRI
- BED increase and OAR doses with MRgSBRT compared to initial ITV-based planning in arms A and C
- collection of treatment plan and irradiation parameters as well as imaging data and quality assurance results for further analysis and planning of follow-up projects, including: evaluation of required adaption frequency and dosimetric benefit of adaption; dosimetric comparison with other radiotherapy techniques; analysis of plan robustness; analysis of the feasibility of MR-only based treatment planning; evaluation and comparisons of different approaches for pseudo-CT generation, auto-contouring, image registration and tracking algorithms; evaluation of fraction dose accumulation methods
- evaluation of potential prognostic biomarkers.

4.3 Explorative Objectives

In a longitudinal evaluation, potential prognostic parameters derived from radiological imaging and blood samples will be evaluated. In particular, plasma levels of hepatic growth factor (HGF) and interleukines (e.g. IL-6 and IL-8) will be evaluated. Tumor genome sequencing will be performed using available samples, e.g. from previous biopsies or surgery, to assess mutations (e.g. KRAS and TP53).

5 Trial Description

5.1 Trial Design

The trial will be performed as a multi-center, three-armed prospective phase II study.

5.2 Trial Duration and Schedule

Planned recruitment time for a total of 82 patients is 36 months. Treatment time will be approximately 1-2 weeks. Patients will be scheduled for follow-up visits at the last day of radiotherapy (or up to one week later), 6 weeks after the end of radiotherapy, three months after

the end of radiotherapy and then every three to six months up to two years of follow-up. Follow-up visits include clinical assessment and contrast-enhanced MRI of the liver.

Total trial duration:	60 months
Recruitment phase:	36 months
Minimal Follow-up:	24 months
FSI (First subject in):	01.02.2021
LSI (Last subject in):	31.01.2024
LSO (Last subject out):	31.01.2026

6 Selection of Patients

6.1 Number of Patients

82 patients will be enrolled in this phase II trial.

6.2 General Criteria for Patients‘ Selection

Patients with hepatic metastases from a primary other than liver will be evaluated and screened based on the protocol. All patients fulfilling the inclusion and exclusion criteria will be informed about the possibility to participate in the study. Registration for the study must be performed prior to beginning of RT.

6.3 Inclusion Criteria

Patients with hepatic metastases of a solid malignancy meeting all of the following criteria will be considered for admission to the trial:

- confirmed underlying solid malignant tumor (no germ cell tumor, leukemia, lymphoma)
- 1-3 hepatic metastases confirmed by pre-therapeutic MRI
- indication for SBRT of 1-3 hepatic metastases
- maximum diameter each hepatic metastasis ≤ 5 cm (in case of 3 metastases: sum of diameters ≤ 12 cm)
- age ≥ 18 years of age
- Karnofsky Performance Score ≥ 60%
- ability to lie still on the radiotherapy treatment couch for at least one hour
- ability to hold one’s breath for more than 25 seconds

- for women with childbearing potential, adequate contraception
- ability of subject to understand character and individual consequences of the clinical trial
- written informed consent (must be available before enrolment in the trial)

6.4 Exclusion Criteria

Patients presenting with any of the following criteria will not be included in the trial:

- refusal of the patients to take part in the study
- patients with primary liver cancer (eg. HCC, CCC)
- patients after liver transplantation
- impairment of liver function to an extent contraindicating radiotherapy (to the discretion of the treating radiation oncologist)
- active acute hepatic/biliary infection (e.g. hepatitis, cholangitis, cholecystitis)
- previous radiotherapy of the hepatobiliary system, if previous and current target volumes overlap
- patients who have not yet recovered from acute toxicities of prior therapies
- claustrophobia
- pregnant or lactating women
- contraindications against performing contrast-enhanced MRI scans (pacemakers, other implants making MRI impossible, allergy to gadolinium (GD)-based contrast agent)
- participation in another competing clinical study or observation period of competing trials

6.5 Criteria for Withdrawal

6.5.1 Withdrawal of Patients

A subject may voluntarily discontinue participation in this study at any time at their own request or at request of the legal representative. In addition, study treatment will be discontinued if unmanageable toxicity is documented, or if the principal investigator decides to terminate the study. A subject will be withdrawn from the protocol if, in the investigator’s opinion, continuation of the trial would be detrimental to the subject’s well-being. If the subject withdraws from the trial and also withdraws consent for disclosure of future information, no further evaluations should be performed, and no additional data should be collected. The PI may retain and continue to use any data collected before such withdrawal of consent, in case the patient has not withdrawn the further use of his/her data as well.

6.5.2 Handling of Withdrawals

In all cases, the reason for withdrawal must be recorded in the Case Report Form (CRF) and in the subject's medical records. In case of withdrawal of a subject at his/ her own request, the reason can be asked and documented. All efforts will be made to follow up the subjects and all examinations scheduled for the final trial day will be performed as far as possible on all patients and documented. All ongoing Adverse Events (AEs)/ Serious Adverse Events (SAEs) of withdrawn patients have to be followed up until no more signs and symptoms are verifiable or the subject is in stable condition.

6.5.3 Premature Withdrawal of Patients from the Study

Individual termination criteria during the treatment phase:

- at any time at the request of the patient
- occurrence of therapy-resistant severe side effects (CTC grade 4 toxicity, which does not recover spontaneously, after supportive therapy or after a radiation break)

6.5.4 Replacement of Patients

Patients will not be replaced if consent is withdrawn retrospectively and patients have already been enrolled.

6.5.5 Individual Termination Criteria during the Follow-up Phase

At any time at the request of the patient.

6.5.6 Premature End of Trial/ Withdrawal of the Whole Study

Reasons for premature termination of the entire study are:

- decision including benefit-risk assessments of the study management when unacceptable risks and toxicities occur
- consideration of termination for each grade 5 toxicity, 2 consecutive grade 4 toxicities, 5 consecutive grade 3 toxicities
- new (scientific) evidence during the study
- inadequate recruitment rate

6.6 Prior and Concomitant Illnesses

Relevant additional illnesses present at the time of informed consent are regarded as concomitant illnesses and will be documented in the patient chart. Abnormalities which appear for the first time

or worsen (intensity, frequency) during the trial are adverse events (AEs) and must be documented on the appropriate pages of the case report form (CRF).

6.7 Prior and Concomitant Treatments

Relevant additional treatments administered to the patients on entry to the trial or at any time during the trial are regarded as concomitant treatments and must be documented on the appropriate pages of the CRF. During radiation therapy, medication required for concomitant illnesses (i.e. hypertension, thyroid disease, hyperlipidemia etc.) can be applied. Concomitant medication should be discussed with the principal investigator on an individual basis. Concomitant systemic therapy or other anti-tumor medication are not part of the study treatment and are only allowed if in accordance with the treating radiation oncologist.

7 MRI

All patients will receive a pre-treatment hepatic MRI for diagnostic and treatment planning purposes. At the study center Heidelberg, specifications of the MRI will be as follows:

- magnetic field strength: 1.5 or 3 Tesla
- contrast agent: Gadolinium-based
- depending on the study visit (screening, T1-T9) the following sequences will be performed:
    - T1 weighted sequences (native and/or contrast-enhanced)
    - T2 weighted sequences (native)
    - diffusion-weighted imaging sequences
    - a TrueFISP sequence in navigator-triggered inspiration
    - additional functional sequences

8 Randomization

After initiation of the study, patients will consecutively be screened and eligible patients will be enrolled into the study. If a BED ≥ 100 Gy is achieved with standard ITV-based planning, patients are randomized to MRgSBRT (arm A) or ITV-SBRT (arm B). If a BED ≥ 100 Gy cannot be achieved with ITV-SBRT planning, the patient will be treated with the highest possible dose using MRgSBRT (arm C). To achieve comparable intervention groups (arm A and B), patients will be allocated in a concealed fashion in a 1:1 ratio by means of randomization using a centralized web-based tool (www.randomizer.at). Randomization will be stratified with respect to the factor centre. Block

randomization with varying block lengths will be performed to achieve in total equal group sizes for randomization.

9 Radiation Therapy

9.1 Treatment Planning and Dose Prescription

Patient eligibility for SBRT will be tested before enrollment in the trial. Training aims to enhance the ability to follow breathing commands including free breathing and deep inspiration breath-hold.

In a first step, treatment planning will be performed with contrast-enhanced and non-enhanced CT scans in free breathing in supine position. Furthermore, patients will undergo a 4D-CT to assess tumor motion in free-breathing. Patients will be immobilized according to standard practice in the specific centre. Based on pre-therapeutic MRI and planning CT, SBRT will be planned using an internal target volume (ITV) concept. Target volume delineation for ITV-based SBRT will be as follows:

- Gross tumor volume (GTV): macroscopic, contrast-enhanced tumor in the planning MR and, if definable, in the planning CT
- Clinical Target Volume (CTV): GTV + 5 mm
- Internal Target Volume (ITV): sum of all GTV contours derived from different breathing phases assessed via 4D-CT
- Planning Target Volume (PTV): ITV + at least 5 mm, depending on the applied image-guidance (e.g. CBCT)

Dose prescription will be performed to the surrounding 65 – 95 % Isodose in up to 10 fractions. For hepatic metastases, α/β =10 is assumed. Dose constraints for OARs are summarized in the following table 1. These dose constraints resemble UK Consensus on Normal Tissue Dose Constraints for Stereotactic Radiotherapy [67]. Further dose constraints of normal tissue will be respected according to German and international guidelines [17, 67, 68].

If a BED of $\geq$ 100 Gy can be achieved with an ITV-concept, patients will be randomized to arms A or B. If a BED of $\geq$ 100 Gy is not achievable using an ITV, patients will be treated with MRgSBRT in arm C without randomization.

In a second step, patients to be treated with MRgSBRT will receive a specific planning CT in deep-inspiration breathhold (non-contrast-enhanced) and simulation at the MR-Linac for MRgSBRT

planning. In the MRgSBRT arms, patients will be immobilized in supine position, and fitted with the MRI receiver coils.

For MRgSBRT (arms A and C), target volume delineation will be as follows:

- Gross tumor volume (GTV): macroscopic, contrast-enhanced tumor in the planning MR and, if definable, in the planning CT
- Clinical Target Volume (CTV): GTV + 5 mm
- Planning Target Volume (PTV): CTV + 3 mm

For MRgSBRT, again, dose prescription will be performed to the surrounding 65 – 95 % Isodose in up to 10 fractions. For hepatic metastases, α/β =10 is assumed. OAR constraints summarized in table 1 must be respected. In arm A, a BED of ≥ 100 Gy must be prescribed. In arm C, patients will be treated via MRgSBRT with the highest achievable dose as deemed by the treating radiation oncologist.

For MRgSBRT (arms A and C), daily OAR contour adaption will be performed within the region PTV_{expand} based on the recommendations by Bohoudi et al. [46]. The PTV_{expand} is the PTV enlarged by 3 cm in transversal and anterior-posterior direction as well as 1 cm in the cranio-caudal direction. The need to adapt the treatment plan will be decided on by the treating physician based on a dose prediction.

For MRgSBRT, real-time cine MR imaging is used for gating. Gating thresholds will be defined daily by the treating physician.

Organ at risk	Dose constraints				
**Number of fractions**	**3 fractions**	**5 fractions**	**6 fractions**	**8 fractions**	**10 fractions**
**Uninvolved liver (=liver-CTV) ≥ 700 cm^3**	< 19.2 Gy	< 24 Gy	< 26 Gy	< 29 Gy	< 32 Gy
**Stomach $D_{0.5cm3}$**	< 22.2 Gy	< 35 Gy	< 37 Gy	< 40 Gy	< 43.5Gy
**Duodenum $D_{0.5cm3}$**	< 22.2 Gy	< 35 Gy	< 37 Gy	< 40 Gy	< 43.5Gy
**Small Bowel $D_{0.5cm3}$**	< 25.2 Gy	< 35 Gy	< 37 Gy	< 40 Gy	< 43.5Gy
**Sigma/Rectum $D_{0.5cm3}$**	< 28.2 Gy	< 34 Gy	< 37 Gy	< 41 Gy	< 44.0 Gy
**Esophagus $D_{0.5cm3}$**	< 25.2 Gy	< 34 Gy	< 36 Gy	< 40 Gy	< 43.5 Gy
**Heart $D_{0.5cm3}$**	< 25.0 Gy	< 29.0 Gy	<3 1.5 Gy	< 60.0 Gy	< 66.0 Gy
**Kidneys Mean dose**	< 8.5 Gy	< 10.0 Gy	< 10.8 Gy	< 11.5 Gy	< 12.0Gy
**Spinal cord $D_{0.1cm3}$**	< 21.6 Gy	< 27.0 Gy	< 29.0 Gy	< 32.0 Gy	< 35.0 Gy
CTV: clinical target volume, D_{xcm3}: dose which is received by less than x cm^3					

Table 1: dose constraints for organs at risk depending on fractionation

9.2 Study Visits

Study visits are also depicted in the flowchart and in table 2. After screening of patients for inclusion and exclusion criteria, successful completion of deep-inspiration breath hold assessment as well as having received informed consent, appropriate patients will be recruited to the trial.
Study relevant data will be collected and patient history will be assessed, including:

- demographic data
- medical history
- physical examination
- comorbidities and concomitant medication
- laboratory evaluation including blood count, liver function parameters and potential biomarkers

The baseline visit (T0) will be scheduled after trial inclusion and prior to the first fraction of SBRT. During the baseline visit (T0) a clinical assessment, as well as analysis of quality of life (using EORTC QLQ C-30 and EORTC QLQ LMC-21) is planned. Baseline symptoms and toxicities will be assessed according to CTCAE V5.0. The patient will need about 30 min to fill out the quality of life questionnaires and for clinical assessment.
Further evaluations will be scheduled during SBRT (mid of treatment, T1), at the last day of treatment (T2), 6 weeks after radiotherapy (T3), every 3 months after radiotherapy during the first year (T4-7) and then every 6 months up to completion of a follow-up of at least 2 years (T8, T9). For patients in the MRgSBRT arms, an in-house developed patient-reported questionnaire, which is already used in the MR-Linac II study (S-862/2019), will be applied after the first fraction of radiotherapy and at the end of radiotherapy to assess acceptance of MR-guided radiotherapy.

- Screening –

Screening for the study will be performed prior to inclusion of the patient into the study. For screening, a planning MRI examination will be performed to assess the number of hepatic lesions as well as their diameter. Furthermore, each patient's ability to perform reproducible phases of breath hold of at least 25 seconds will be evaluated to assess tolerability of breath-hold-gated RT. All inclusion and exclusion criteria must be fulfilled.

- Baseline Visit (T0)

Baseline Visit will be performed after inclusion of the patient into the study and prior to the first fraction of SBRT.

The following examinations will be performed:

- clinical assessment using CRF
- quality of life using the EORTC QLQ-C30, EORTC QLQ LMC-21
- MR-Linac-specific questionnaire in arms A and C
- toxicity according to CTCAE V5.0
- laboratory evaluation including blood count, liver function parameters and potential biomarkers

-Study Visit (T1)

The first study visit is scheduled at mid of treatment.

The following examinations will be performed:

- diagnostic, non-contrast enhanced MRI (at study site Heidelberg only)
- laboratory evaluation including blood count, liver function parameters and potential biomarkers

-Study Visit (T2)

The second study visit is scheduled at the last day of radiotherapy.

The following examinations will be performed:

- clinical assessment using CRF
- assessment of adverse events
- quality of life using the EORTC QLQ-C30, EORTC QLQ LMC-21
- toxicity according to CTCAE V5.0
- MR-Linac-specific questionnaire in arms A and C
- diagnostic, non-contrast enhanced MRI (at study site Heidelberg only)
- laboratory evaluation including blood count, liver function parameters and potential biomarkers

-Study Visit (T3)

The third study visit is scheduled 6 weeks (+/- 1 week) after end of treatment. The following examinations will be performed:

- clinical assessment using CRF
- assessment of adverse events
- quality of life using the EORTC QLQ-C30, EORTC QLQ LMC-21

- toxicity according to CTCAE V5.0
- diagnostic, contrast enhanced MRI (+/- 7 days before/after T3)
- laboratory evaluation including blood count, liver function parameters and potential biomarkers

-Study Visits during first year of follow-up (T4,5,6,7)

The fourth and later study visits are scheduled every 3 months (+/- 4 weeks) after end of treatment, i.e. 3, 6, 9 and 12 months after end of treatment. The following examinations will be performed:

- clinical assessment using CRF
- assessment of adverse events
- quality of life using the EORTC QLQ-C30, EORTC QLQ LMC-21
- toxicity according to CTCAE V5.0
- contrast-enhanced MRI (+/- 14 days before/after Tn)
- laboratory evaluation including blood count, liver function parameters and potential biomarkers

-Study Visits during the second year of follow-up (T8,9)

In the second year of follow-up, study visits are scheduled every 6 months (+/- 4 weeks) after end of treatment, i.e. 18 and 24 months after end of treatment. The following examinations will be performed:

- clinical assessment using CRF
- assessment of adverse events
- quality of life using the EORTC QLQ-C30, EORTC QLQ LMC-21
- toxicity according to CTCAE V5.0
- contrast-enhanced MRI (+/- 14 days before/after Tn)

Study-related time investment for each patient is estimated to be about 20-30 minutes per follow-up visit (completion of CRFs).

	Screening	Baseline (before SBRT)	Mid of SBRT	End of SBRT	Follow-up		
					6 weeks after SBRT	1st year after SBRT:	2nd year after SBRT:

						every 3 months	every 6 months
		T0	T1	T2	T3	T4-7	T8-9
Medical history		x		x	x	x	x
EORTC QLQ-C30 and LMC21		x		x	x	x	x
MR-Linac questionnaire (only treatment arm A)		x		x			
Documentation of medication		x		x	x	x	x
Documentation of AEs		x		x	x	x	x
CRF form		x		x	x	x	x
Blood test		x	x	x	x	x	
Breathhold assessment (› 25 seconds?)	x						
MRI (c=contrast enhanced, n=without)	x (c)		x (n)	x (n)	x (c)	x (c)	x (c)

10 Trial Methods

The primary objective is to demonstrate non-inferiority of MRgSBRT to ITV-SBRT regarding treatment-related hepatobiliary and gastrointestinal toxicity during the first year following SBRT according to CTCAE V5.0.

Secondary analyses include comparing all three treatment groups with respect to the primary endpoint, the number of lesions in which a BED of ≥ 100 Gy can be achieved (yes / no), potential BED increase of ≥ 5 Gy using MRgSBRT compared to ITV-SBRT, treatment-related toxicity, quality of life, local, locoregional and distant tumor control, progression-free and overall survival and morphological and functional changes in MRI. Additionally, treatment plans and irradiation

parameters as well as imaging data will be collected for quality assurance results for further analysis and planning of follow-up projects.

10.1 Assessment of Efficacy Parameters

10.1.1 Primary Endpoint: non-inferiority of MRgSBRT to ITV-SBRT regarding treatment-related toxicity

The primary endpoint is defined as the occurrence of treatment-related gastrointestinal and hepatobiliary CTCAE V5.0 toxicity of grade III or higher. The primary aim of the study is the assessment of non-inferiority of MRgSBRT to ITV-SBRT regarding the primary endpoint, assuming a non-inferiority margin of $\delta = 10\%$. Detailed acute and chronic, potentially therapy-related toxicity will be assessed during each follow-up visit and documentation of side-effects will be performed with Common Terminology Criteria for Adverse Events and with the Radiation Therapy Oncology Group (RTOG)/ European Organization for Research and Treatment of Cancer (EORTC) Late Radiation Morbidity Scoring System Scheme. Toxicity assessment will especially focus on all grades of gastrointestinal and hepatobiliary toxicity and will be assessed according to CTCAE V5.0.

10.1.2 Secondary Endpoints

Plan Quality

Plans will be compared in terms of biologically effective dose, total dose, single doses, prescribed isodoses, dose homogeneity as well as doses to organs at risk (e.g. stomach, small bowel, duodenum, liver, esophagus, heart, lungs, kidneys, spinal cord). In particular, the number of lesions in which a BED of ≥ 100 Gy can be achieved (yes / no) and potential BED increases of ≥ 5 Gy using MRgSBRT for patients in arms A and C will be assessed. Furthermore, conformity will be analyzed and compared as previously described by the modified Paddick Cornformity Index [69, 70].

Local Tumor Control

Defined as the number of days between randomization until diagnosis of local progression of the irradiated metastasis within the high-dose area, which is defined within 1 cm surrounding the PTV. Tumor recurrence or progression within this area is defined as local failure, while death is considered as a competing event.

Locoregional Tumor Control

Defined as the number of days between randomization until tumor recurrence or progression in the liver, more than 1 cm from the PTV, is defined as locoregional progression. Locoregional failure is not similar to treatment failure. Death is considered as a competing event.

Distant Tumor Control

Distant tumor control is defined as number of days from randomization until occurrence of distant extrahepatic metastases, death without prior distant progression, or end of follow-up. For patients alive and not diagnosed with distant progression at the end of the study, the distant tumor control time will be censored at the time of the last study visit.

Progression-free Survival

Progression-free survival defined as number of days from randomization until the first occurrence of local, locoregional or distant tumor recurrence or progression, tumor-related death, death without prior progression, or end of follow-up. For patients alive and not diagnosed with progression at the end of the study, the disease-free survival time will be censored at the time the patient was last known to be free of progression of tumor disease.

Overall Survival

Overall survival time, defined as number of days from randomization until death or end of follow-up. For patients alive at the end of the study, the overall survival time will be censored at the time of the last visit or follow-up contact.

Quality of Life (QoL)

QoL will be analyzed with the help of the validated 30-item self-assessment questionnaire of the European Organization for Research and Treatment of Cancer (EORTC QLQ-C30, version 3.0). It is composed of five multi-item functional scales (physical, role, emotional, cognitive, and social function), three multi-item symptom scales (fatigue, pain, nausea/vomiting) combined with a global health and quality-of life scale. The other six single items assess further symptoms (dyspnea, insomnia, appetite loss, constipation and diarrhea) that are often reported by cancer patients as well as financial difficulties [71]. Scores are interpreted according to the guidelines of the EORTC Scoring Manual [72]. The QLQ-C30 questionnaire will be complemented with the colorectal liver cancer module of the EORTC (QLQ- LMC-21), initially designed for patients with colorectal liver metastases [73, 74]. QLQ-LMC-21 comprises another 21 items in five scales (nutritional, fatigue, pain, emotional) and six single items assessing further symptoms (weight, loss of taste, xerostomia, oral mucositis, paresthesia, jaundice).

Furthermore, for patients treated with MRgSBRT (arm A and C), patient-reported acceptance of the procedure will be evaluated using an in-house developed patient-reported outcome questionnaire, which has already been used in the MR-Linac II study of the Department of Radiation Oncology, University Hospital Heidelberg (S-862/2019). The questionnaire will be completed after the first fraction and after the last fraction. The MR-Linac questionnaire consists of questions regarding potential MR-related experiences and complaints (e.g. noise, bore size, fixation with coils). Furthermore, the perception of their active role during gated dose delivery SBRT is assessed. Items are scored using a 5-point scale. Furthermore, patients are asked to report the time they need to recover from the procedure after the first fraction. MR-Linac staff will further document the patient's compliance on a 0-10 scale after the first and last fraction.

Morphological and Functional Changes in MRI

MRI including morphological and functional sequences will be analyzed for the purpose of changes in treated metastases and surrounding normal tissue. Qualitative and quantitative changes will be evaluated.

Acquisition and comparison of different MRI sequences

Different MRI sequences will be acquired and assessed for comparability and/or superiority. Quantitative and qualitative differences will be analyzed.

Technical Analyses of Treatment

The treatment plan and irradiation parameters as well as imaging and quality assurance data derived within this study are acquired pseudonymously for further analysis and planning of follow-up projects:

The required adaption frequency and resulting dosimetric benefit is evaluated, comparing the daily adapted treatments plans with the non-adapted plans in terms of dosimetric parameters (e.g. target volume coverage, minimum, maximum and mean dose within the target volume and organs at risk). Moreover, the plan re-optimization robustness is analyzed. Therefore, it is monitored if and how the optimization parameters have to be changed to obtain clinical acceptable plans calculated on the daily MR images.

Based on the planning MR and CT as well as the daily MR images, re-plannings for different treatment techniques and adaption scenarios are performed and compared using e.g. dosimetric plan parameters as well as required beam times.

Furthermore, several concepts of pseudo-CT generation based on the daily MR data are tested. Clinical treatment plans calculated based on the planning CT electron density are re-calculated on pseudo-CTs generated from the MR image and dosimetric differences are analyzed by means of e.g. minimum, mean and maximum dose within the target volume and organs at risk as well as dose-volume-parameters. Based on these results, the technical and dosimetric feasibility of an MR-only workflow is assessed. Treatment plans calculated based on the sole information of the daily MR-image are compared to clinical plans and the required time for treatment planning as well as for the entire treatment of both workflows are analyzed. Moreover, quality assurance results like e.g. plan integrity checks and dosimetric measurements results are evaluated.

Additionally, the daily MR-images are employed to test automatic contour and image registration tools. For this purpose, automatically generated contours are compared to manually drawn contours using similarity measures (e.g. DICE coefficients, location and volume of contours). In order to assess different image registration algorithms, the generated vector fields are evaluated and the location and volumes of anatomical structures within the registered images are compared.

Besides, different tracking methods are analyzed using the daily MR-images. A target structure is contoured in a reference image and tracked in the following images via an automatic algorithm. The automatically tracked structure is compared to manually generated contours of the target structure in terms of location and shape.

Moreover, different dose accumulation methods of individually adapted treatment fractions are evaluated. Based on the daily MR-images as well as daily dose volumes, the total dose is summed and dose differences are analyzed.

Assessment of potential biomarkers

Analyses include cytokinetic assays to assess tissue remodeling (e.g. CTGF, TGFβ, VEGF, PDGF), inflammation (e.g. CCL2, CXCL12 / SDF1, G (M) CSF) and quantification of immunocytokines (e.g. IL-6, IL-13, IL-17, IL-33, IL-10, TNF-α) before SBRT, mid-treatment, at the end of treatment and during the first year of follow-up.
Further, available tumor samples (e.g. after resection or biopsy of the primary, circulating tumor cells from blood draws) will be analyzed for mutations (e.g. BRAF, EGFR, HER2, KRAS, NRAS, PIK3CA, TP53).

11 Plan for Treatment or Care after the Trial

After completion of study treatment, any standard treatment may be considered. Any systemic treatment or chemotherapy is not part of the clinical trial. For tumor progression, treatment alternatives will be evaluated and discussed interdisciplinarily considering options of surgical resection, systemic therapy (e.g. chemotherapy, molecular targeted therapies, immunotherapy), further local therapies (e.g. RFA, SIRT, TACE) or hepatic SBRT of newly diagnosed liver metastases.

12 Assessment of Safety

During and following a subject’s participation in the trial, the investigator should ensure that adequate medical care is provided to a subject for any adverse event. The investigator should inform a subject when medical care is needed for intercurrent illness(es) of which the investigator becomes aware. RT will be carried out as ambulatory treatment; however, admission to the ward for supportive care is possible when necessary. Supportive measures including skin care and pain management will be performed at the discretion of the treating radiation oncologist.

12.1 Adverse Events

Toxicity associated to irradiation that occurs for the first time after treatment start or preexisting toxicity that significantly increases in severity grade after the start of study treatment likely to be related to radiotherapy will be documented within the subject’s medical records and eCRF. Treatment related side effects will be recorded according to CTCAE v5.0 (Common Terminology Criteria for Adverse Events version 5.0; http://www.eortc.be/services/doc/ctc). Grades refer to the severity of the adverse event.

Acute toxicities are defined by the occurrence within the first 90 days after the start of RT. Adverse Events occurring after the first 90 days until 6 months after study treatment will be documented as subacute toxicities. Any adverse event emerging more than 6 months after RT will be recorded as late toxicity.

Standardized informed consent forms for irradiation of hepatic metastases are utilized to inform the patient about study treatment. Expected acute side effects of RT include radiation dermatitis, fatigue, pain, nausea, diarrhea, colitis and enteritis, loss of appetite. Characteristics and severity of subacute/ late side effects depend on the location and the extent of the target volume/ irradiation field and include skin fibrosis, lung fibrosis, rib fractures/osteonecrosis, soft tissue necrosis, cardiac insufficiency, coronary sclerosis, arrhythmia, decreased liver function, RILD, kidney malfunction, infections, perforation of hollow organs, gastrointestinal ulcera, gastrointestinal bleeding, peripheral nerve disorders, spinal cord toxicity (e.g. paraplegia).

In detail:

- skin disorders: radiation dermatitis, dryness and pruritus of the skin, alopecia
- gastrointestinal disorders: nausea, inappetence, emesis, colitis, diarrhea, obstipation, meteorism, liver enzyme elevations, liver function disorders, RILD, gastritis, colitis, enteritis, cholecystitis, cholangitis, gastric/small bowel/colon perforation, fistulae, ulcers
- kidney disorders
- infections, hepatitis b reactivation
- rib fractures/osteonecrosis, soft tissue necrosis/fibrosis
- hot flashes, night sweats, bone pain, fever, weight loss
- bleedings, thromboses (e.g. of the portal vein)
- pain
- lung disorders: coughing, dyspnea, fibrosis, pneumonitis
- heart disorders: coronary sclerosis, arrhythmia, cardiac insufficiency
- sensomotoric disorders

12.2 Serious Adverse Events

A serious adverse event is an adverse event with a special degree of severity in that it

- leads to death (CTCAE grade 5)
- is life-threatening (CTCAE grade 4) due to its actual and documented severity at the point of occurrence (not only life-threatening in principle if occurrence was more severe)
- causes or prolongs hospitalization

- leads to a lasting or otherwise significant impairment
- is an otherwise significant medical incident

The following examples are not to be considered SAE:

- Medical or surgical interventions
- Hospital admissions that are not the consequence of a medical condition (e. g. social/ convenience admissions)
- day-to-day fluctuations of pre-existing illness(es) or toxicity present or detected at the start of the study that do not worsen
- A planned hospitalization where admission did not take longer than anticipated
- Tumor progression and all medical interventions performed to offer respective therapy or relieve the symptoms of such progression

13 Statistical Methods

13.1 Analysis Populations

Full Analysis Set (FAS): All patients who fulfilled the inclusion and exclusion criteria and hence were included into the trial. The term “intention-to-treat (ITT)-analysis” is used for an analysis applying ITT principles to all patients of the FAS. This will be the primary analysis set for the primary endpoint.

Per Protocol Set (PP): All patients from the FAS, excluding patients with major protocol violations. Major protocol violations will be discussed with the coordinating investigator.

Safety Set: All patients from the FAS who received at least one fraction of radiotherapy. Patients will be allocated to the treatment they actually received.

The allocation of each patient to the different analysis populations will be defined and explained in further detail in the statistical analysis plan (SAP).

13.2 Sample Size Calculation

The sample size calculation is based on the primary comparison of the rates of gastrointestinal and hepatobiliary toxicity CTCAE of grade III or higher between the two randomized treatment groups (arm A and B). A total of 62 patients are needed to assess non-inferiority by means of a Farrington-Manning test of MRgSBRT to ITV- SBRT with a power of 80% at a one-sided significance level of 10%, with allowance for 5% loss to follow-up and with the use of a clinically relevant non-

inferiority margin of δ =10%, when assuming toxicity rates of pMRgSBRT=2% and pITV-SBRT=5%. It is expected that using a shifted Mantel-Haenszel type test for non-inferiority adjusting for the factor centre will yield an increased power.

To achieve a comparable arm C (BED ‹ 100 Gy) for the secondary objectives, the study will recruit patients until at least 20 analysable patients are present in arm C. Due to the fact that patients in arm C are still treated with the best possible treatment, this approach is ethically justifiable.

Sample size calculation was performed using PASS 16.0.3.

13.3 Statistical Analysis

13.3.1 Primary Endpoint

The primary analysis will be based on the FAS including all enrolled patients according to the intention to treat principle. The hypotheses for the primary analysis are

$$H_0{:}\, p_{\mathrm{MRgSBRT}} - p_{\mathrm{ITV-SBRT}} \geq \delta \quad vs.\ H_1{:}\, p_{\mathrm{MRgSBRT}} - p_{\mathrm{ITV-SBRT}} < \delta$$

(δ =10% non-inferiority margin), where p_{MRgSBRT} and $p_{\mathrm{ITV-SBRT}}$ are the probabilities for an occurrence of a gastrointestinal and hepatobiliary toxicity CTCAE of grade III or higher for the MRgSBRT (arm A) and the ITV-SBRT (arm B) group, respectively. Non-inferiority of MRgSBRT as compared to ITV-SBRT will be tested at an one-sided significance level of α=0.1 using a Mantel-Haenszel type test for non-inferiority adjusting for the stratum "centre". Missing values for the primary outcome will be imputed using multiple imputation [75]. Sensitivity analyses will be performed by means of conducting an analysis for the per-protocol (PP) population (based on those patients without major protocol violation).

13.3.2 Secondary Endpoints

Analysis of the secondary endpoints will also be based on the FAS. In order to compare all three treatment groups with regard to the primary endpoint, a (descriptive) Chi-squared test will be used.

The secondary endpoints distant tumor control, overall survival and progression-free survival will be analyzed using Kaplan-Meier-Curves. The 1-year and 2-year survival rates as well as the median survival rate will be provided alongside two-sided 95%- confidence intervals. Descriptive pairwise logrank tests stratified for "centre" will be conducted to compare all three treatment groups. The secondary endpoints local and locoregional control will be analyzed via cumulative incidence functions, taking the competing event death into account.

Patients treated with MRgSBRT will be assessed with regard to the rate of lesions in which a BED increase of ≥ 5 Gy can be achieved compared to ITV-SBRT. This will be achieved by conducting a (descriptive) McNemar test.

The other secondary endpoints and the patients` characteristics will be displayed by descriptive measures. Descriptive pairwise comparisons between all three treatment groups will be performed. Continuous variables will be described using number non-missing values, mean, standard deviation, median, Q1, Q3, minimum and maximum. For binary or categorical variables absolute and relative frequencies will be provided. Furthermore, two-sided 95%- confidence intervals will be calculated.

13.3.3 Safety Analysis

The safety analysis is based on the safety set including all patients who received one of the study treatments, and includes calculation of frequencies and rates of adverse and serious adverse events together with corresponding 95%-confidence intervals.

Further details of the analysis will be specified in the statistical analysis plan (SAP) which will be finalized before database closure. All analyses will be done using SAS version 9.4 or higher.

14 Quality Assurance

14.1 SOP (Standard Operating Procedures)

All participating investigators are guided by the study-specific SOPs that are provided by the study administration.

14.2 Data Quality

To ensure data quality and consistency, internal quality control measures are carried out. For this purpose, at least 10% of all patients included up to this time (selected at random) are monitored twice a year by an internal monitor within the framework of quality assurance (internal monitoring). Monitoring includes:

- Fulfillment of the inclusion criteria,
- treatment according to the study protocol (treatment arm, medical treatment and treatment planning),
- follow-ups according to the protocol (time points, diagnosis, evaluation of tumor size and side effects),
- review of regulatory documentation and notification of AEs / SAEs.

- verification of recruitment and drop-out rates.
- verification of documentation in the HIRO (Heidelberger Institut für Radioonkologie) database
- verification of keeping study cockpit (“Studiencockpit”) up to date and complete

After each internal monitoring, the principal investigator receives a monitoring report. This is kept in the investigator site file (ISF).

15 Documentation

15.1 Data Management

The data is collected, managed and processed electronically in the in-house Heidelberg Institute of Radiation Oncology (HIRO) research database. It is the responsibility of the principal investigator to conduct the study in accordance with applicable legal provisions and the study protocol, and that the data is entered correctly and completely in the electronic case report forms (eCRFs). All data collected in this study must be documented by authorized persons in the eCRFs. Access to the database must be authorized in writing by the principal investigator (signature log). Access authorization may not be passed to third parties.

Data in the HIRO database will be checked by programmed value ranges, validity and consistency checks. If necessary, queries may arise that are made using the HIRO database and authorized persons. Based on the queries, the study physician / study nurse can review and answer or correct the resulting discrepancies.

After completion of the study and after entry of all relevant data and clarification of the queries, the data base will be closed. The originals of all central study documents, including documentation sheets, are kept at the Study Center for at least 15 years after the final report has been prepared.

15.2 Patient Identification List

Study participation is recorded by registering the patient within the study cockpit (“Studiencockpit”) of the clinic's hospital information system in an electronic patient identification list. It is used exclusively by the study personnel for the subsequent identification of the participating patients. This list is kept absolutely confidential and archived for at least 15 years after the end of the study. In addition, the patient’s participation in this clinical trial is marked in the patient record.

15.3 Investigator Site File (ISF)

The Investigator Site File stores the documents required for the clinical study. The principal investigator is responsible for keeping the ISF up to date and complete. After completion or termination of the study, it must be kept for at least 15 years.

15.4 Data Storage

The originals of all central study documents, including CRFs, are kept in the study center for at least 15 years after the final report has been prepared. The principal investigator keeps the administrative documents (correspondence with the ethics committee, study administration, study center), the patient identification list, the signed informed consent forms, copies of the CRFs and the general study documentation (protocol, amendments) for the above mentioned time. Original data of study patients (medical records) must be kept for at least 15 years.

16 Reports, Publications

16.1 Final Report

All information pertaining to this clinical trial should be treated confidentially. The statistical analysis and the preparation of a final report will be realized and signed by the principal investigator, the study coordinator and biometrician within 12 months after the closure of the database. All information contained in this report is strictly confidential.

16.2 Publications

For the international publication of the study results, this study protocol was registered in the database of the National Institute of Health (www.clinicaltrials.gov) or in the German Register of Clinical Trials (DRKS) (https://www.drks.de/drks_web/).

The results of this clinical study will be published under the responsibility of the principal investigator. Regarding the primary study endpoint, the first and last authorship are reserved for the principal investigator and the primary study coordinator if both do not wish to transfer their authorship to a third person. All information about the primary study endpoint must be kept confidential until then. The final publication is planned after the end of the study. The presentation of the results in the context of a publication is based on existing requirements for publications.

Data and research concerning radiological aspects can and shall be published under the respective radiology department.

17 Ethical, Legal and Administrative Aspects

17.1 Responsibilities of the Principal Investigator

The principal investigator is responsible for initiating, organizing and funding the clinical trial. The clinical trial is carried out in accordance with the existing laws and regulations, in accordance with the current version of the Declaration of Helsinki and the provisions of the Radiation Protection Ordinance (Strahlenschutzverordnung) and the Radiation Protection Law (Strahlenschutzgesetz). The recommendations of Good Clinical Practice (see ICH-GCP: International Conference on Harmonization - Good Clinical Practice, in the latest version) are taken into account (http://www.ema.europa.eu/docs/en_GB/document_ library/Scientific_ guideline/ 2009/09/WC500002874.pdf).

The present study is neither a clinical trial of drugs under the German Medicines Act (Arzneimittelgesetz, AMG) nor a medical device according to the Medical Devices Act (Medizinproduktegesetz, MPG). The present study can be carried out in accordance with the requirements of § 23b MPG as a non-MPG non-AMG study ("Sonstige Studie"), since the MRIdian Linac from ViewRay acc. §§ 6 and 10 of the MPG is entitled to carry the CE mark. The study is not aiming for conformity assessment of the CE mark and no additional invasive or other stressful examinations are performed.

The responsibilities of the principal investigator at the study center are amongst others:

- Ensuring that all persons involved in the study are adequately informed about the study protocol, any changes to the study protocol, the treatment plan and its study-specific tasks and functions,
- ensuring that sufficient time and capacity are available to conduct the study,
- correct collection and documentation of the data,
- ensuring that the information about participants and all information is treated confidentially by all persons involved in the study,
- maintaining a list of all study physicians or other suitably qualified personnel to whom essential study-specific tasks have been delegated.

17.2 Ethics Committee, DEGRO Expert Committee

Study protocol, patient information and the informed consent form are submitted to the ethics committee of the Medical Faculty of the University of Heidelberg for professional counseling. The study will start only after receiving the approval. The Ethics Committee will be promptly informed by the principal investigator of any changes in the study protocol that may affect patient safety. In the clinical study, no ionizing radiation in humans for the purpose of medical research according to § 31 StrlSchG is used, since all treatments within this protocol are clinically indicated and performed within the medical responsibility of the participating centers. An application to the Federal Office for Radiation Protection (Bundesamt für Strahlenschutz, BfS) is therefore not required. The study was submitted to the DEGRO expert committee for advice. Recruitment will not start before the committee classifies the protocol as a medical science ("Heilkunde").

17.3 Patient Information and Informed Consent

After informing the patient - in oral and written form - of the nature, significance, implications, expected benefits and potential risks of the clinical trial, each patient must provide written informed consent to participate in the study before enrollment. The patient must be provided with sufficient time and opportunity to decide on her participation prior to the initiation of any study measures and to be able to clarify open questions with the attending physician. The informed consent form includes the date and signature of the participant and the study physician. A copy of the informed consent form and the patient information will be handed out to the participant; the original will be placed in the Investigator Site File.

Furthermore, the patient has the option to decide separately on the transfer of his data to third parties, a refusal has no effect on study participation and on the further use of his data outside the aims of this study (e.g. meta-analyses).

The study participant can withdraw the consent at any time and without stating reasons. The study participant is asked to give the reason for withdrawal, but it is pointed out that he or she does not have to do this. The information about the withdrawal must be documented in the patient file as well as on the participant's informed consent form. On request, a copy of the correspondingly amended informed consent form will be handed out. The treating physician/ study nurse must ensure that the revocation of consent is communicated to the data management. The datasets will remain on the in-house HIRO database and may continue to be used for scientific studies. In the case of consent withdrawal, (data-) material that has already been obtained is destroyed or the patient is asked whether he or she agrees to the evaluation of the material.

17.4 Patient Insurance

Since only clinically established therapies and diagnostics are used within the study, there is no study-specific insurance. As for treatments outside of studies, this means that study participants are not insured for the health damage or other adverse effects that they might experience in connection with participation in this study at the University Hospital Heidelberg, unless the physician or his staff meets culpable misconduct. Intent and negligence are to be regarded as culpable misconduct.

The study participants are not accident insured on the way to and from, as well as during an outpatient irradiation, as all visits would also take place as normal follow-up visits beyond this study.

17.5 Data Protection and Medical Confidentiality

The names of the patients and all other confidential information are subject to medical confidentiality and the provisions of the General Data Protection Regulation (DSGVO) as well as “Landesdatenschutzgesetz” and “Bundesdatenschutzgesetz” (LDSG or BDSG). Patient data will only be shared in pseudonymized form. Third parties do not get any insight into original documents. The prerequisite for this is the voluntary approval of the study participants in informed consent form. For this, the study participants are informed about the following:

1. Personal data collected in the context of this clinical study, in particular health and ethnic information, will be recorded in paper form and electronically in case report forms (CRFs) at the radiotherapy clinic.
2. Authorized and confidential staff of the Department of Radiation Oncology and Radiotherapy, University Hospital Heidelberg can view personal data for monitoring. To ensure the quality of the study, the data may be transferred in pseudonymized form to authorized representatives of the leading study center. For this measure, the study physician is released from his medical confidentiality.
3. The collected data, including imaging material, will be exported in pseudonymized form for scientific research purposes in the field of cancer research in collaboration with other institutions (possibly with private companies or partners abroad with possibly lower data protection levels). For research purposes, the pseudonymized data may be linked to other

data from other sources (such as diagnostic records, treatment planning, cancer registry, medical records, etc.).

4. The consent can be withdrawn by the patient at any time, without giving reasons and without disadvantages for further medical care. In the case of such a revocation of the consent, the data may continue to be used, as long as there is no request for the complete deletion of the data.
5. Health data of the study participant can be collected or viewed by co-treating physicians as necessary for the proper conduct and monitoring of the study. In that regard, physicians are released from confidentiality.
6. The study participant may allow that his or her family physician / other treating physicians are informed about his or her participation in the study and may be asked for further information as part of the follow-up.

18 Funding

An application for financial support for the MAESTRO study by the Dietmar Hopp Foundation has been submitted and has been granted. All persons involved (including the principal investigator and co-investigators) declare that there is no conflict of interest in connection with the implementation and evaluation of this study.

19 Amendments

In the interests of sound data analysis, changes in the study protocol are not scheduled. In exceptional cases, however, changes to the study conditions are possible. Any change to the study procedure must be made in writing, stating the reasons, and signed by all persons responsible for the study. The changes will then be considered part of the study protocol. If required (e.g., dose changes of the radiation and / or other significant changes that directly affect the safety of the study participants), the approval of the responsible ethics committees and the study participant must be obtained. Changes or additions to the study protocol can only be initiated and authorized by the principal investigator.

20 Signatures

The present trial protocol was subject to critical review and has been approved in the present version by the persons undersigned. The information contained is consistent with:

- the current risk-benefit assessment of the investigational treatment
- the moral, ethical, and scientific principles governing clinical research as set out the principles of GCP and in the applicable version of Declaration of Helsinki. The investigator will be supplied with details of any significant or new finding including AEs relating to treatment with the investigational treatment.

PD Dr. Juliane Hörner-Rieber, Principal Investigator
Radiation Oncology
Date: Signature

________________ ________________________

Prof. Dr. Dr. Jürgen Debus, Co-PI
Radiation Oncology
Date: Signature

26.11.2021 ________________________

Dr. Philipp Hoegen, Principal study coordinator
Radiation Oncology
Date: Signature

________________ ________________________

Prof. Dr. Heinz-Peter Schlemmer, Study coordinator
Radiology
Date: Signature

________________ ________________________

Dr. Johannes Krisam, Trial biometrician
Institute of Medical Biometry and Informatics

Date: Signature

References:

1. Adam, R., et al., *Hepatic resection for noncolorectal nonendocrine liver metastases: analysis of 1,452 patients and development of a prognostic model.* Ann Surg, 2006. **244**(4): p. 524-35.
2. House, M.G., et al., *Survival after hepatic resection for metastatic colorectal cancer: trends in outcomes for 1,600 patients during two decades at a single institution.* J Am Coll Surg, 2010. **210**(5): p. 744-52, 752-5.
3. Nordlinger, B., et al., *Perioperative FOLFOX4 chemotherapy and surgery versus surgery alone for resectable liver metastases from colorectal cancer (EORTC 40983): long-term results of a randomised, controlled, phase 3 trial.* The Lancet Oncology, 2013. **14**(12): p. 1208-1215.
4. Tomlinson, J.S., et al., *Actual 10-Year Survival After Resection of Colorectal Liver Metastases Defines Cure.* Journal of Clinical Oncology, 2007. **25**(29): p. 4575-4580.
5. Smith, J.J. and M.I. D'Angelica, *Surgical management of hepatic metastases of colorectal cancer.* Hematol Oncol Clin North Am, 2015. **29**(1): p. 61-84.
6. Cummings, L.C., J.D. Payes, and G.S. Cooper, *Survival after hepatic resection in metastatic colorectal cancer: a population-based study.* Cancer, 2007. **109**(4): p. 718-26.
7. Borgelt, B.B., et al., *The palliation of hepatic metastases: results of the Radiation Therapy Oncology Group pilot study.* Int J Radiat Oncol Biol Phys, 1981. **7**(5): p. 587-91.
8. Bydder, S., et al., *A prospective trial of short-fractionation radiotherapy for the palliation of liver metastases.* Australas Radiol, 2003. **47**(3): p. 284-8.
9. Munoz-Schuffenegger, P., S. Ng, and L.A. Dawson, *Radiation-Induced Liver Toxicity.* Seminars in Radiation Oncology, 2017. **27**(4): p. 350-357.
10. Pan, C.C., et al., *Radiation-associated liver injury.* Int J Radiat Oncol Biol Phys, 2010. **76**(3 Suppl): p. S94-100.
11. Toesca, D.A.S., et al., *Strategies for prediction and mitigation of radiation-induced liver toxicity.* J Radiat Res, 2018. **59**(suppl_1): p. i40-i49.
12. Lee, U.E. and S.L. Friedman, *Mechanisms of hepatic fibrogenesis.* Best Pract Res Clin Gastroenterol, 2011. **25**(2): p. 195-206.
13. An, S.Y., A.D. Petrescu, and S. DeMorrow, *Targeting Certain Interleukins as Novel Treatment Options for Liver Fibrosis.* Front Pharmacol, 2021. **12**: p. 645703.
14. Dawson, L.A., et al., *Analysis of radiation-induced liver disease using the Lyman NTCP model.* Int J Radiat Oncol Biol Phys, 2002. **53**(4): p. 810-21.
15. Chang, D.T., et al., *Stereotactic body radiotherapy for colorectal liver metastases: a pooled analysis.* Cancer, 2011. **117**(17): p. 4060-9.
16. Rusthoven, K.E., et al., *Multi-institutional phase I/II trial of stereotactic body radiation therapy for liver metastases.* J Clin Oncol, 2009. **27**(10): p. 1572-8.
17. Sterzing, F., et al., *Stereotactic body radiotherapy for liver tumors: principles and practical guidelines of the DEGRO Working Group on Stereotactic Radiotherapy.* Strahlenther Onkol, 2014. **190**(10): p. 872-81.
18. Yamashita, H., et al., *Local effect of stereotactic body radiotherapy for primary and metastatic liver tumors in 130 Japanese patients.* Radiation Oncology, 2014. **9**(1): p. 112.
19. Doi, H., et al., *Stereotactic Body Radiation Therapy for Liver Tumors: Current Status and Perspectives.* Anticancer Res, 2018. **38**(2): p. 591-599.
20. Ohri, N., et al., *Local Control After Stereotactic Body Radiation Therapy for Liver Tumors.* Int J Radiat Oncol Biol Phys, 2018.
21. Doi, H., et al., *Threshold doses and prediction of visually apparent liver dysfunction after stereotactic body radiation therapy in cirrhotic and normal livers using magnetic resonance imaging.* J Radiat Res, 2016. **57**(3): p. 294-300.
22. Kavanagh, B.D., et al., *Radiation dose-volume effects in the stomach and small bowel.* Int J Radiat Oncol Biol Phys, 2010. **76**(3 Suppl): p. S101-7.
23. Miften, M., et al., *Radiation Dose-Volume Effects for Liver SBRT.* Int J Radiat Oncol Biol Phys, 2018.
24. Bae, S.H., et al., *Predictor of Severe Gastroduodenal Toxicity After Stereotactic Body Radiotherapy for Abdominopelvic Malignancies.* International Journal of Radiation Oncology*Biology*Physics, 2012. **84**(4): p. e469-e474.
25. Bertholet, J., et al., *Time-Resolved Intrafraction Target Translations and Rotations During Stereotactic Liver Radiation Therapy: Implications for Marker-based Localization Accuracy.* Int J Radiat Oncol Biol Phys, 2016. **95**(2): p. 802-9.

26. Poulsen, P.R., et al., *Kilovoltage intrafraction motion monitoring and target dose reconstruction for stereotactic volumetric modulated arc therapy of tumors in the liver.* Radiother Oncol, 2014. **111**(3): p. 424-30.
27. Worm, E.S., et al., *Variations in magnitude and directionality of respiratory target motion throughout full treatment courses of stereotactic body radiotherapy for tumors in the liver.* Acta Oncol, 2013. **52**(7): p. 1437-44.
28. Hof, H., et al., *4D-CT-based target volume definition in stereotactic radiotherapy of lung tumours: Comparison with a conventional technique using individual margins.* Radiotherapy and Oncology, 2009. **93**(3): p. 419-423.
29. Worm, E.S., et al., *Fiducial marker guided stereotactic liver radiotherapy: Is a time delay between marker implantation and planning CT needed?* Radiotherapy and Oncology, 2016. **121**(1): p. 75-78.
30. Kan, M.W., et al., *Radiation dose from cone beam computed tomography for image-guided radiation therapy.* Int J Radiat Oncol Biol Phys, 2008. **70**(1): p. 272-9.
31. Lincke, T. and C.J. Zech, *Liver metastases: Detection and staging.* Eur J Radiol, 2017. **97**: p. 76-82.
32. Khoo, V.S. and D.L. Joon, *New developments in MRI for target volume delineation in radiotherapy.* Br J Radiol, 2006. **79 Spec No 1**: p. S2-15.
33. Bonekamp, D., et al., *Association of overall survival in patients with newly diagnosed glioblastoma with contrast-enhanced perfusion MRI: Comparison of intraindividually matched T1 - and T2 (*) - based bolus techniques.* J Magn Reson Imaging, 2015. **42**(1): p. 87-96.
34. Donaldson, S.B., et al., *Perfusion estimated with rapid dynamic contrast-enhanced magnetic resonance imaging correlates inversely with vascular endothelial growth factor expression and pimonidazole staining in head-and-neck cancer: a pilot study.* Int J Radiat Oncol Biol Phys, 2011. **81**(4): p. 1176-83.
35. Yu, J.I., et al., *The role of diffusion-weighted magnetic resonance imaging in the treatment response evaluation of hepatocellular carcinoma patients treated with radiation therapy.* Int J Radiat Oncol Biol Phys, 2014. **89**(4): p. 814-21.
36. Lambrecht, M., et al., *Value of diffusion-weighted magnetic resonance imaging for prediction and early assessment of response to neoadjuvant radiochemotherapy in rectal cancer: preliminary results.* Int J Radiat Oncol Biol Phys, 2012. **82**(2): p. 863-70.
37. d'Assignies, G., et al., *High sensitivity of diffusion-weighted MR imaging for the detection of liver metastases from neuroendocrine tumors: comparison with T2-weighted and dynamic gadolinium-enhanced MR imaging.* Radiology, 2013. **268**(2): p. 390-9.
38. Surov, A., H.J. Meyer, and A. Wienke, *Correlation between apparent diffusion coefficient (ADC) and cellularity is different in several tumors: a meta-analysis.* Oncotarget, 2017. **8**(35): p. 59492-59499.
39. Acharya, S., et al., *Online Magnetic Resonance Image Guided Adaptive Radiation Therapy: First Clinical Applications.* Int J Radiat Oncol Biol Phys, 2016. **94**(2): p. 394-403.
40. Noel, C.E., et al., *Comparison of onboard low-field magnetic resonance imaging versus onboard computed tomography for anatomy visualization in radiotherapy.* Acta Oncol, 2015. **54**(9): p. 1474-82.
41. Boldrini, L., et al., *Use of Indirect Target Gating in Magnetic Resonance-guided Liver Stereotactic Body Radiotherapy: Case Report of an Oligometastatic Patient.* Cureus, 2018. **10**(3): p. e2292.
42. Henke, L., et al., *Phase I trial of stereotactic MR-guided online adaptive radiation therapy (SMART) for the treatment of oligometastatic or unresectable primary malignancies of the abdomen.* Radiother Oncol, 2018. **126**(3): p. 519-526.
43. Li, X.A., et al., *Interfractional Variations in Patient Setup and Anatomic Change Assessed by Daily Computed Tomography.* International Journal of Radiation Oncology*Biology*Physics, 2007. **68**(2): p. 581-591.
44. Wysocka, B., et al., *Interfraction and Respiratory Organ Motion During Conformal Radiotherapy in Gastric Cancer.* International Journal of Radiation Oncology*Biology*Physics, 2010. **77**(1): p. 53-59.
45. Henke, L.E., et al., *Stereotactic MR-Guided Online Adaptive Radiation Therapy (SMART) for Ultracentral Thorax Malignancies: Results of a Phase 1 Trial.* Adv Radiat Oncol, 2019. **4**(1): p. 201-209.
46. Bohoudi, O., et al., *Fast and robust online adaptive planning in stereotactic MR-guided adaptive radiation therapy (SMART) for pancreatic cancer.* Radiotherapy and Oncology, 2017. **125**(3): p. 439-444.
47. Henke, L.E., et al., *Magnetic Resonance Image-Guided Radiotherapy (MRIgRT): A 4.5-Year Clinical Experience.* Clin Oncol (R Coll Radiol), 2018. **30**(11): p. 720-727.

48. Rosenberg, S.A., et al., *A Multi-Institutional Experience of MR-Guided Liver Stereotactic Body Radiation Therapy.* Adv Radiat Oncol, 2019. **4**(1): p. 142-149.
49. Feldman, A.M., et al., *Real-time Magnetic Resonance-guided Liver Stereotactic Body Radiation Therapy: An Institutional Report Using a Magnetic Resonance-Linac System.* Cureus, 2019. **11**(9): p. e5774.
50. Klüter, S., et al., *First prospective clinical evaluation of feasibility and patient acceptance of magnetic resonance-guided radiotherapy in Germany.* Strahlenther Onkol, 2020.
51. Tetar, S., et al., *Patient-reported Outcome Measurements on the Tolerance of Magnetic Resonance Imaging-guided Radiation Therapy.* Cureus, 2018. **10**(2): p. e2236.
52. Hong, T.S., et al., *Phase II Study of Proton-Based Stereotactic Body Radiation Therapy for Liver Metastases: Importance of Tumor Genotype.* J Natl Cancer Inst, 2017. **109**(9).
53. Ajdari, A., et al., *Toward Personalized Radiation Therapy of Liver Metastasis: Importance of Serial Blood Biomarkers.* JCO Clin Cancer Inform, 2021. **5**: p. 315-325.
54. Dawood, O., A. Mahadevan, and K.A. Goodman, *Stereotactic body radiation therapy for liver metastases.* European Journal of Cancer, 2009. **45**(17): p. 2947-2959.
55. Fischer-Valuck, B.W., et al., *Two-and-a-half-year clinical experience with the world's first magnetic resonance image guided radiation therapy system.* Adv Radiat Oncol, 2017. **2**(3): p. 485-493.
56. Mutic, S. and J.F. Dempsey, *The ViewRay System: Magnetic Resonance–Guided and Controlled Radiotherapy.* Seminars in Radiation Oncology, 2014. **24**(3): p. 196-199.
57. Herfarth, K.K., et al., *Extracranial stereotactic radiation therapy: set-up accuracy of patients treated for liver metastases.* Int J Radiat Oncol Biol Phys, 2000. **46**(2): p. 329-35.
58. Herfarth, K.K., et al., *Stereotactic Single-Dose Radiation Therapy of Liver Tumors: Results of a Phase I/II Trial.* Journal of Clinical Oncology, 2001. **19**(1): p. 164-170.
59. Herfarth, K.K., et al., *[Stereotactic irradiation of liver metastases].* Radiologe, 2001. **41**(1): p. 64-8.
60. Andratschke, N., et al., *The SBRT database initiative of the German Society for Radiation Oncology (DEGRO): patterns of care and outcome analysis of stereotactic body radiotherapy (SBRT) for liver oligometastases in 474 patients with 623 metastases.* BMC Cancer, 2018. **18**(1): p. 283.
61. Habermehl, D., et al., *Single-dose radiosurgical treatment for hepatic metastases - therapeutic outcome of 138 treated lesions from a single institution.* Radiation Oncology, 2013. **8**(1): p. 175.
62. Herfarth, K.K., J. Debus, and M. Wannenmacher, *Stereotactic radiation therapy of liver metastases: update of the initial phase-I/II trial.* Front Radiat Ther Oncol, 2004. **38**: p. 100-5.
63. Herfarth, K.K., et al., *Assessment of focal liver reaction by multiphasic CT after stereotactic single-dose radiotherapy of liver tumors.* International Journal of Radiation Oncology*Biology*Physics, 2003. **57**(2): p. 444-451.
64. Klement, R.J., et al., *Stereotactic body radiotherapy for oligo-metastatic liver disease – Influence of pre-treatment chemotherapy and histology on local tumor control.* Radiotherapy and Oncology, 2017. **123**(2): p. 227-233.
65. Network, N.C.C. *NCCN Clinical Practice Guidelines in Oncology (NCCN Guidelines®) Colon Cancer.* Version 4.2018 18.02.2019]; Available from: https://www.nccn.org/professionals/physician_gls/pdf/colon.pdf.
66. Network, N.C.C. *NCCN Clinical Practice Guidelines in Oncology (NCCN Guidelines®) Rectal Cancer.* Version 3.2018 2018 18.02.2019]; Available from: https://www.nccn.org/professionals/physician_gls/pdf/rectal.pdf.
67. Hanna, G.G., et al., *UK Consensus on Normal Tissue Dose Constraints for Stereotactic Radiotherapy.* Clin Oncol (R Coll Radiol), 2018. **30**(1): p. 5-14.
68. Guckenberger, M., et al., *Definition of stereotactic body radiotherapy: principles and practice for the treatment of stage I non-small cell lung cancer.* Strahlenther Onkol, 2014. **190**(1): p. 26-33.
69. Audet, C., et al., *Evaluation of volumetric modulated arc therapy for cranial radiosurgery using multiple noncoplanar arcs.* Med Phys, 2011. **38**(11): p. 5863-72.
70. Paddick, I., *A simple scoring ratio to index the conformity of radiosurgical treatment plans. Technical note.* J Neurosurg, 2000. **93 Suppl 3**: p. 219-22.
71. Aaronson, N.K., et al., *The European Organization for Research and Treatment of Cancer QLQ-C30: a quality-of-life instrument for use in international clinical trials in oncology.* J Natl Cancer Inst, 1993. **85**(5): p. 365-76.
72. Treatment, E.O.f.R.a.C. *EORTC QLQ-C30 Scoring Manual*

2001; 3rd:[Available from: https://www.eortc.be/qol/files/SCManualQLQ-C30.pdf.

73. Kavadas, V., et al., *Development of an EORTC disease-specific quality of life questionnaire for use in patients with liver metastases from colorectal cancer.* Eur J Cancer, 2003. **39**(9): p. 1259-63.
74. Blazeby, J.M., et al., *Validation of the European Organization for Research and Treatment of Cancer QLQ-LMC21 questionnaire for assessment of patient-reported outcomes during treatment of colorectal liver metastases.* Br J Surg, 2009. **96**(3): p. 291-8.
75. van Buuren, S., *Multiple imputation of discrete and continuous data by fully conditional specification.* Stat Methods Med Res, 2007. **16**(3): p. 219-42.
